# Induction of mitochondrial dysfunction as a strategy for targeting tumour cells in metabolically compromised microenvironments

**DOI:** 10.1038/ncomms4295

**Published:** 2014-02-18

**Authors:** Xiaonan Zhang, Mårten Fryknäs, Emma Hernlund, Walid Fayad, Angelo De Milito, Maria Hägg Olofsson, Vladimir Gogvadze, Long Dang, Sven Påhlman, Leoni A. Kunz Schughart, Linda Rickardson, Padraig D′Arcy, Joachim Gullbo, Peter Nygren, Rolf Larsson, Stig Linder

**Affiliations:** 1 Department of Oncology-Pathology, Karolinska Institute, Stockholm S-171 76, Sweden; 2 Division of Clinical Pharmacology, Department of Medical Sciences, Uppsala University, Uppsala S-751 85, Sweden; 3 Division of Toxicology, Institute of Environmental Medicine, Karolinska Institutet, Stockholm 171 77, Sweden; 4 Division of Hematology/Oncology, Department of Internal Medicine, University of Florida Shands Cancer Center, University of Florida, Gainesville, Florida 32601, USA; 5 Department of Laboratory Medicine, Center for Molecular Pathology, CREATE Health, Skåne University Hospital, Lund University, S-20502, Malmö, Sweden; 6 OncoRay—National Center for Radiation Research in Oncology, TU Dresden, Dresden 01307, Germany; 7 Division of Oncology, Department of Radiology, Oncology and Radiation Science, Uppsala University, Uppsala S-751 85, Sweden; 8 These authors contributed equally to this work; 9 Present address: In vitro Bioassay Laboratory, Pharmacognosy Department, National Research Center, Dokki, Giza 12622, Egypt

## Abstract

Abnormal vascularization of solid tumours results in the development of microenvironments deprived of oxygen and nutrients that harbour slowly growing and metabolically stressed cells. Such cells display enhanced resistance to standard chemotherapeutic agents and repopulate tumours after therapy. Here we identify the small molecule VLX600 as a drug that is preferentially active against quiescent cells in colon cancer 3-D microtissues. The anticancer activity is associated with reduced mitochondrial respiration, leading to bioenergetic catastrophe and tumour cell death. VLX600 shows enhanced cytotoxic activity under conditions of nutrient starvation. Importantly, VLX600 displays tumour growth inhibition *in vivo*. Our findings suggest that tumour cells in metabolically compromised microenvironments have a limited ability to respond to decreased mitochondrial function, and suggest a strategy for targeting the quiescent populations of tumour cells for improved cancer treatment.

## Abstract (web summary)

Quiescent sub-populations of cells in tumours are resistant to traditional chemotherapeutics and are responsible for tumour recurrence. Here, Zhang *et al*. identify a compound that kills quiescent tumour cells in solid tumour tissue by inducing mitochondrial dysfunction.

## Graphical abstract

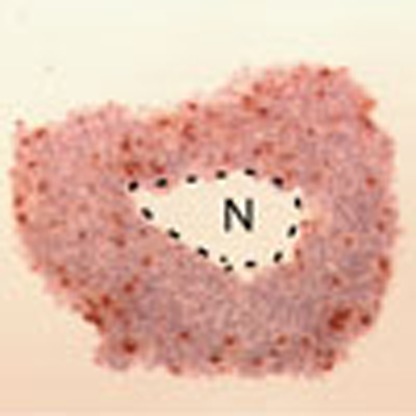

A fundamental problem in cancer drug discovery is to identify compounds that eliminate dormant malignant cells responsible for tumour relapse. Abnormal vascularization of solid tumours leads to the generation of tissue microenvironments that are chronically starved of oxygen and nutrients^1^,^2^. Cells residing in such environments are slowly growing or quiescent and display altered phenotypic characteristics when compared with cells located in more vascularized regions^3^,^4^,^5^. Such non-dividing cells are often resistant to mainstay standard chemotherapies that rely on DNA replication and cell division to elicit their antitumour effect^6^. The altered phenotype of quiescent cells enables them to survive chemotherapeutic regimes and reseed nascent tumours following secession of chemotherapy^7^. Indeed, resistance to agents such as doxorubicin, cisplatin and vinblastine has been correlated with poor vasculature, tumour relapse and poor patient survival^8^. Thus, there is a need to alter the scope of cancer drug discovery and focus more on screening for agents that can exploit the altered phenotype of quiescent metabolically stressed cells to eliminate tumours.

Cell-based screening for novel anticancer drugs is typically performed using monolayer cultures of tumour cells^9^; however, such monolayer cultures do not represent the characteristics of three-dimension (3-D) solid tumours, frequently leading to the failure of subsequent *in vivo* models. The multicellular tumour spheroid model is of intermediate complexity between *in vivo* tumours and *in vitro* monolayer cultures and is more suitable for drug screening and evaluation^10^. Spheroids are known to be more resistant to drug effects compared with monolayer cultures^11^. Resistance is not only due to pharmacokinetic obstacles limiting drug penetrance to inner layers but also due to multicellular interactions leading to altered expression of genes and proteins regulating drug response^10^,^12^,^13^. An additional benefit of growing cells three-dimensionally is the opportunity to explore the core regions’ potential vulnerability related to hypoxia and nutrient deficiency and to reflect the heterogenous milieu in tumour microregions. Poorly vascularized and perfused tumour microareas in many aggressive cancers have limited access not only to oxygen but also to glucose^14^,^15^. Core regions are also associated with acidic pH (Acker *et al*.^16^) since these tumour cells change their metabolism towards increased glycolysis, resulting in increased lactic acid production.

Here we employ spheroid cultures of HCT116 colon cancer cells to screen a diverse chemical library with the aim to find compounds with cytotoxic activity in core, hypoxic, regions. The screen identified a compound (VLX600) demonstrating anticancer activity with a large therapeutic window both *in vitro* and *in vivo.* We characterize the mechanism of action and potential of VLX600 as anticancer therapy. Our findings show that VLX600 decreases mitochondrial oxidative phosphorylation (OXPHOS), and suggests that tumour cells in metabolically compromised microenvironments are unable to respond to decreased mitochondrial function.

## Results

### VLX600 reduces multicellular spheroid viability

**Figure 1:**
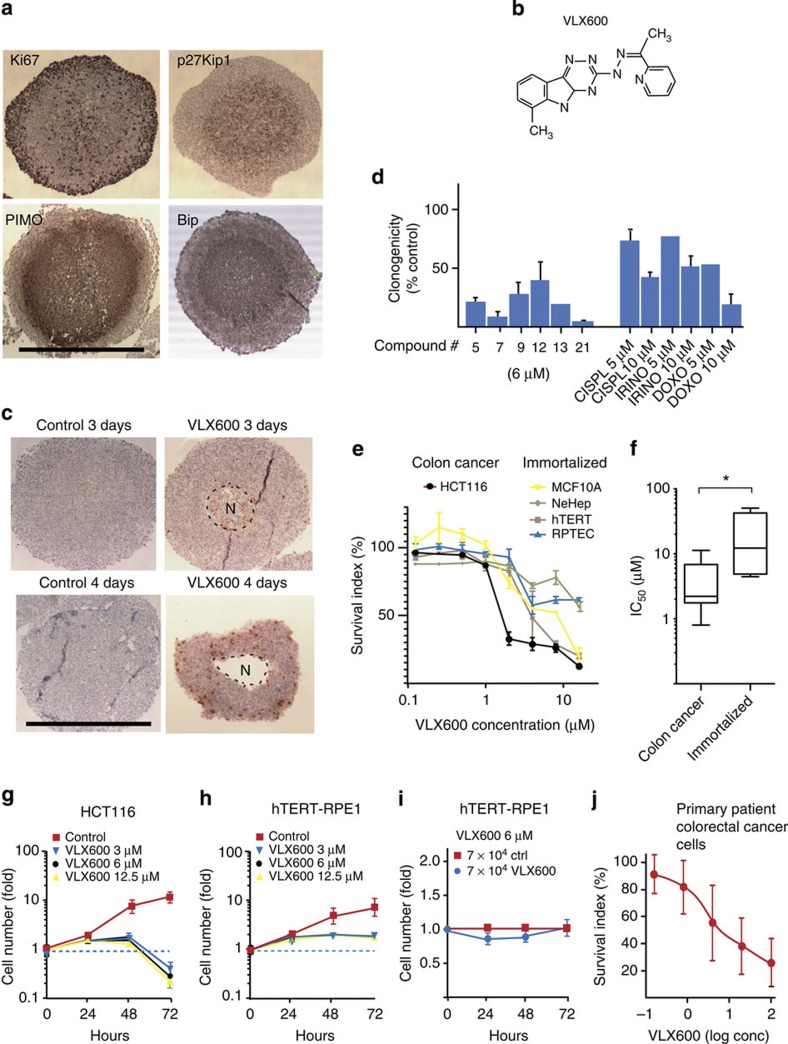
VLX600 is cytotoxic to HCT116 spheroids and has a therapeutic window. (**a**) Sections of HCT116 colon cancer MCS (5 days after seeding cells in hanging drops), stained for Ki67, p27^Kip1^, pimonidazole adducts or Bip/Grp78. Scale bar, 500 μm. (**b**) Structure of VLX600. (**c**) HCT116 MCS were exposed to 6 μM VLX600 for 6 h and further incubated in drug-free medium, sectioned and stained for active caspase-3. Note the smaller size and necrotic areas (‘N’) of treated MCS. Median sections, scale bar, 500 μm. (**d**) VLX600 induces loss of clonogenicity of HCT116 MCS. Spheroids were exposed to each compound for 6 h, incubated for 5 days, dispersed and seeded for clonogenic outgrowth. Compound 21=VLX600 (*n*=3; means±s.d.). (**e**) Monolayer cultures of HCT116 cells and four immortalized cell lines were exposed to VLX600 for 72 h and viability determined using the fluorometric microculture cytotoxicity assay test^58^; (*n*=3; means±s.e.m.). (**f**) Different sensitivities of colon cancer and normal cells to VLX600. The IC_50_ values of colon cancer cells (HCT116, HT29, SW620, HT8, DLD and RKO) and immortalized cells (hTERT-RPE1, MCF10A, RPTEC/TERT1 and NeHepLxHT) are presented as box-plots (medians, 25–75th percentiles) and whiskers (10–90th percentiles; *P<0.05; the Mann–Whitney test). (**g**) Proliferation of HCT116 monolayer cells in the presence or absence of VLX600. Cells were seeded at 7,000 cells per well in 96-well plates. Viability was determined by the acid phosphatase test; note the decrease in cell number between 48 and 72 h. Shown are means±s.d. (*n*=3). (**h**) Proliferation of immortalized hTERT-RPE1 monolayer cells in the presence or absence of VLX600, conditions as in **e**, means±s.d. (*n*=3). (**i**) hTERT-RPE1 cells were seeded at 70,000 cells per well in 96-well plates in the presence or absence of 6 μM VLX600. hTERT-RPE1 cells do not proliferate at this cell density, VLX600 does not affect cell viability. Shown are means±s.d. (*n*=3). (**j**) Survival of primary tumour cells from colon carcinoma patients at different concentrations of VLX600; incubation was for 72 h (*n*=22). Shown are means±s.d.

To identify compounds that are active against quiescent tumour cell populations, HCT116 colon carcinoma multicellular spheroids (MCS) were used as targets for a 10,000 compound drug screen as described^17^. As shown in Fig. 1a, HCT116 cells grown as MCS consist of a peripheral region of cells expressing the proliferation marker Ki67 encapsulating a non-proliferating quiescent core expressing high levels of the cyclin-dependent kinase inhibitor p27^Kip1^. Comparison of Gene Set Enrichment Analysis (GSEA) of microarray data^18^ between HCT116 cells grown as monolayers and MCS showed upregulation of genes associated with hypoxia and glycolysis concomitant with downregulation of mitosis-associated genes (Supplementary Fig. 1a). Hypoxia was confirmed by staining for pimonidazole adducts (Fig. 1a). Cells in MCS core regions stained positively for BiP/Grp78 (Fig. 1a), an endoplasmic reticulum (ER) chaperone. Two other markers of ER stress were observed in MCS, eIF2-α phosphorylation and caspase-4 activation (Supplementary Fig. 1b). Similar to solid tumour tissue^15^,^19^,^20^, and consistent with previous reports^21^,^22^, MCS contain decreased levels of glucose per cell (Supplementary Fig. 1c). Furthermore, glucose was found to be an essential nutrient for MCS viability since decreasing glucose concentrations in the culture medium resulted in death of cells in inner areas (Supplementary Fig. 1d). We conclude that HCT116 colon carcinoma MCS contain populations of quiescent and stressed cells known to be resistant to various forms of cancer therapy^23^,^24^,^25^. Furthermore, our data show that glucose is necessary for core cell viability and that glucose diffusion is limited in MCS.

Spheroids were exposed to drugs for 6 h and reduction of viability was determined after an additional 72 h incubation in a drug-free medium. VLX600 (ChemBridge cpd 5625138; ChemBridge, San Diego, CA; Fig. 1b) was the most active of ~10,000 compounds screened (for details on the screen, see Supplementary Fig. 1e). VLX600 exposure (6 μM) resulted in the appearance of central necrotic areas (‘N’) after 72–96 h and modest induction of active caspase-3 in stained sections (Fig. 1c). Importantly, exposure of MCS to VLX600 resulted in a strong decrease in clonogenicity of dispersed cells (Fig. 1d).

VLX600 inhibited the proliferation of HCT116 (Fig. 1e), five additional human colon carcinoma cell lines and two mouse cell lines (Supplementary Fig. 1f) in monolayer culture over 72 h. A sub-population of the cells was partially resistant to the compound. A significant difference in the sensitivities to VLX600 (IC_50_ values) between colon cancer and immortalized cells was observed (Fig. 1e,f). We also examined the response of HCT116 colon cancer cells and immortalized human telomerase reverse transcriptase-retinal pigment epithelial 1 (hTERT-RPE1) cells to VLX600 over time. HCT116 cells were growth arrested at 48 h, followed by a decrease in cell numbers indicating cell death (Fig. 1g, Supplementary Fig. 1g). Cell death showed features of apoptosis (Supplementary Fig. 1h–k); however, overall survival was not affected by caspase inhibitors (Supplementary Fig. 1l), suggesting an apoptotic independent mechanism of cell death. In contrast to the cancer cell lines, immortalized epithelial hTERT-RPE1 cells became growth arrested in the absence of detectable cytotoxicity (Fig. 1h; Supplementary Fig. 1g). Furthermore, when non-proliferating, confluent hTERT-RPE1 cells were exposed to VLX600, no loss of cell viability was observed (Fig. 1i). These results suggest that VLX600 displays selective cytotoxic activity against malignant cells.

We also examined the ability of VLX600 to inhibit the proliferation of primary cultures of patient colorectal cancer cells *ex vivo* (*n*=22; Fig. 1j). Interestingly, the VLX600 concentrations required to reduce cell survival of *ex vivo* patient cells was significantly below those achievable in rodent plasma following intravenous injection (see below), suggesting the possibility of achieving clinical relevant drug exposure.

### Induction of hypoxic and glycolytic responses by VLX600

**Figure 2:**
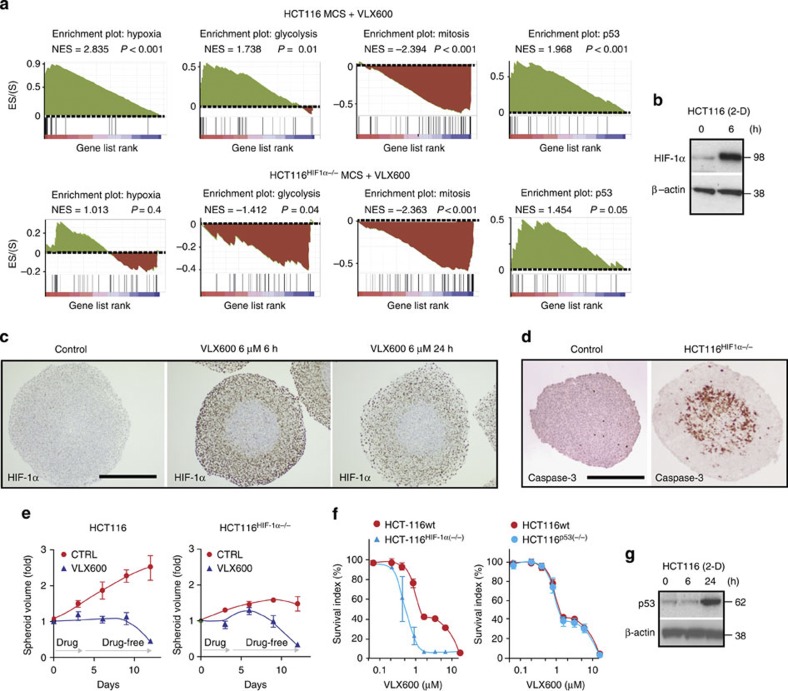
Induction of HIF-1α and a glycolytic response by VLX600. (**a**) GSEA plots of hypoxia, glycolysis, mitosis and p53 networks based on the gene expression profiles of HCT116 MCS or HCT116^HIF-1α−/−^ MCS exposed for 6 h to 6 μM VLX600. NES, normalized enrichment score; *P* values refer to the nominal *P* values generated after 1,000 permutations. (**b**) Monolayer HCT116 cells were exposed to 6 μM VLX600 and analysed for HIF-1α expression by western blotting after 6 h. (**c**) Spheroids were exposed to VLX600 for different times, sectioned and stained for HIF-1α. The spheroid core areas were lack of HIF-1α induction. Scale bar, 250 μm (**d**) HCT116^HIF-1α−/−^ MCS show strong positivity for active caspase-3 in core areas. Spheroids were fixed and stained for active caspase-3 5 days after formation; scale bar, 250 μm. (**e**) HCT116 and HCT116^HIF-1α−/−^ MCS were exposed for 72 h to VLX600 (6 μM) followed by drug wash-out. Diameters of the MCS were recorded and converted to volumes. Volume determinations are inaccurate at 4–10 days since MCS disintegrate and dead cells stick to spheroid structures (the transient increase in the volume of VLX600-treated HCT116^HIF-1α−/−^ MCS is likely to be an artifact). Shown are means±s.d. (*n*=3). (**f**) HCT116, HCT116^HIF-1α−/−^ or HCT116^p53−/−^ monolayer cells were exposed to VLX600 and viability was determined after 72 h using the fluorometric microculture cytotoxicity assay. Shown are means±s.e.m. (*n*=3). (**g**) Monolayer HCT116 cells were exposed to 6 μM VLX600 and analysed for p53 expression by immunoblotting at the times indicated.

HCT116 MCS were exposed to VLX600 or vehicle for 6 h followed by microarray-based gene expression analysis. GSEA of genes induced by VLX600 showed a strong positive correlation to genes associated with hypoxia, glycolysis and genes regulated by p53, and a negative correlation to genes associated with mitosis (Fig. 2a). Consistent with the induction of a hypoxic gene signature, expression of the hypoxia-inducible factor (HIF)-1α transcription factor was upregulated by VLX600 in monolayer cells (Fig. 2b) at the promoter level (Supplementary Fig. 2a). HIF-1α was induced in the outer layers of MCS but not in the inner core cells (Fig. 2c). Since VLX600 affected the viability (Fig. 1c) and the phenotype of cells in the MCS core areas, the lack of HIF-1α induction in core areas is unlikely to be due to limited penetration of the drug. Rather, the absence of HIF-1α induction may be explained by HIF-1α not being stabilized under conditions of low glucose availability^26^. The upregulation of genes associated with hypoxia and glycolysis, as well as increased cellular production of lactate, was dependent on HIF-1α (Fig. 2a; Supplementary Fig. 2b). We conclude that VLX600 induces a HIF-1α-dependent glycolytic response.

We examined whether HIF-1α has a cell survival role in drug-exposed cells. Consistent with previous reports^27^, core areas of HCT116^HIF-1α−/−^ MCS contained numerous apoptotic cells (Fig. 2d), and these MCS grew slower than their HCT116^wt^ counterparts. HCT116^HIF-1α−/−^ MCS were sensitive to VLX600 (Fig. 2e) and HCT116^HIF-1α−/−^ monolayer cells were, in fact, more sensitive than parental cells (Fig. 2f). The partially VLX600-resistant cell population observed in HCT116 and other colon cancer cell lines (somewhat varying between experiments) was not observed in the HCT116^HIF-1α−/−^ cells. We conclude that HIF-1α partially protects against VLX600-induced cell death. However, since HIF-1α is not induced in MCS cores, cells in these regions are likely to display increased sensitivity to the drug.

We finally examined the potential role of the p53 response to VLX600. Induction of p53 was confirmed by western blotting (Fig. 2g); however, disruption of the *p53* gene did not affect the sensitivity of HCT116 cells to VLX600 (Fig. 2f).

### VLX600 induces an autophagic response

**Figure 3:**
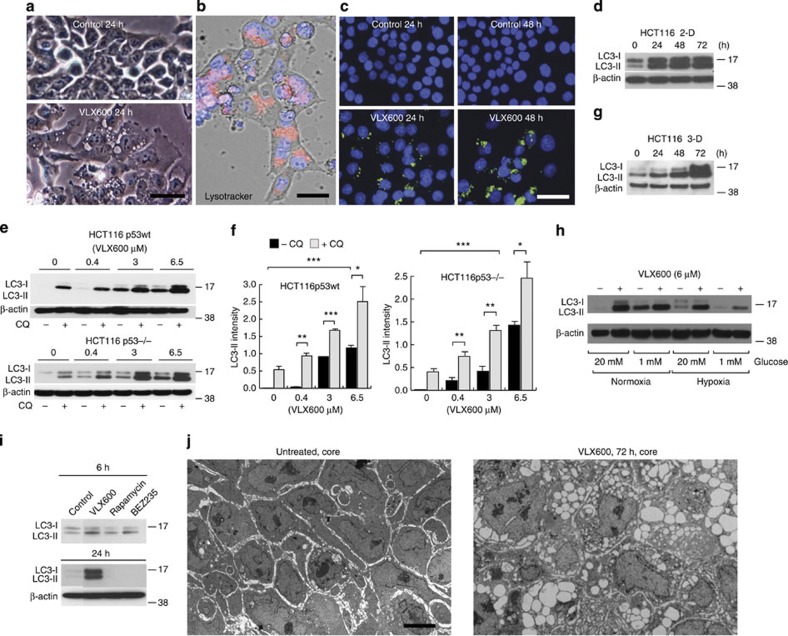
Induction of autophagy by VLX600. (**a**) Phase contrast microphotographs of monolayer HCT116 cells exposed to 6 μM VLX600 for 24 h showing cytoplasmic vacuoles (scale bar, 20 μm). (**b**) Acidic vacuoles in HCT116 cells exposed to 6 μM VLX600 for 24 h revealed by staining with Lysotracker (scale bar, 10 μm). (**c**) HCT116 cells were exposed to VLX600 (6 μM) and stained with an antibody to LC3 (scale bar, 20 μm). (**d**) Time-dependent induction of LC3-II protein by 6 μM VLX600 in HCT116 monolayer cultures. Extracted proteins were subjected to immunoblotting. (**e**) VLX600 induces autophagic flux in monolayer HCT116 and HCT116^p53−/−^ cells. Proteins were extracted from cells exposed to different concentrations of VLX600 in the presence or absence of 25 μM chloroquine (CQ) and subjected to immunoblot analysis. Short film exposures were used to allow detection of the effect of CQ. (**f**) LC3-II levels were quantified relative to β-actin (shorter exposure films were used for scanning). Treatments as in **e**. Shown are means±s.e.m. (*n*=3); (*P<0.05, **P<0.01, ***P<0.001; *t*-test). (**g**) Induction of LC3-II protein by 6 μM VLX600 in MCS cultures of HCT116. Proteins were extracted and subjected to immunoblotting. (**h**) Induction LC3-II protein by 6 μM VLX600 in monolayer HCT116 cells during conditions of high and low glucose, and normoxia and hypoxia (0.5% O_2_). LC3 was detected by immunoblotting. (**i**) Sustained induction of LC3-II is observed after treatment with VLX600 but not with rapamycin or NVP-BEZ235 (an mTOR inhibitor and PI3K/mTOR inhibitor). Cells were treated for the times indicated and processed for immunoblotting (β-actin control for 24 h panel). (**j**) Electron micrographs of MCS sections. HCT116 MCS were treated with 0.5% DMSO (left) or 6 μM VLX600 (right) for 6 h at day 5 after formation. MCS were further incubated for 72 h sectioned and processed for EM. Scale bar, 5 μm.

Before cell death, VLX600-exposed HCT116 cells increased in size and showed the presence of large cytoplasmic vesicles (Fig. 3a). These vesicles stained positively with Lysotracker (Fig. 3b), suggesting them to be autolysosomes. Positive staining was also observed using an antibody to microtubule-associated protein 1 light chain 3 (LC3; Fig. 3c), but not with an antibody to the ER marker calnexin (Supplementary Fig. 3a). Already at 3 h exposure, an increase in LC3-positive cytoplasmic dots, indicating autophagosomes, was observed in exposed cells (Supplementary Fig. 3b). Autophagosomes containing cellular material were observed by electron microscopy (Supplementary Fig. 3c). LC3-II (the phosphatidylethanolamine-conjugated form of LC3), an autophagosome marker^28^, was induced by VLX600 in HCT116 cells in monolayer cultures over 72 h (Fig. 3d). LC3-II was induced in a dose-dependent manner, independently of p53 expression (Fig. 3e,f). The levels of LC3-II were further increased by co-exposure with the lysosomotropic agent chloroquine (Fig. 3e,f; Supplementary Fig. 3d), consistent with an increased autophagic flux in VLX600-exposed cells. Autophagy was confirmed by reduced levels of p62/SQSTM1 (Bjorkoy *et al*.^29^; Supplementary Fig. 3e). Spheroids showed elevated basal levels of LC3-II expression (Supplementary Fig. 3f), which was further stimulated by VLX600 (Fig. 3g). Interestingly, LC3-II induction by VLX600 was similar at high and low glucose conditions, suggesting that a maximal capacity for autophagy induction was reached (Fig. 3h; Supplementary Fig. 3g). We conclude that VLX600-induced cell death is associated with induction of autophagy.

Interruption of mammalian target of rapamycin (mTOR) signalling is known to stimulate autophagy^30^. We indeed found that VLX600 inhibited phosphorylation of the mTOR downstream effectors 4EBP1 and p70-S6K by an HIF-1α-independent mechanism (Supplementary Fig. 3h,i). The strong and sustained induction of LC3-II could not, however, be mimicked by the mTOR inhibitor rapamycin or by the PI3K/mTOR inhibitor NVP-BEZ235 (Fig. 3i). Furthermore, the cytotoxic effect of VLX600 on MCS could not be mimicked by NVP-BEZ235 (Supplementary Fig. 3k,l). We conclude that, although mTOR signalling is inhibited by VLX600, neither induction of autophagy nor cytotoxicity can be explained by inhibition of mTOR signalling.

Since vesicle formation is a morphological marker of VLX600 exposure, we examined the occurrence of vesicles in HCT116 MCS after transient exposure to VLX600. Examination of sections by electron microscopy revealed massive vacuolization in cells in the cores of the MCS (Fig. 3j). We conclude that VLX600 induces an autophagic response and induction of autolysosomes in HCT116 cells; importantly, this response was observed in spheroid core regions.

### Autophagy protects against a bioenergetic catastrophe

**Figure 4:**
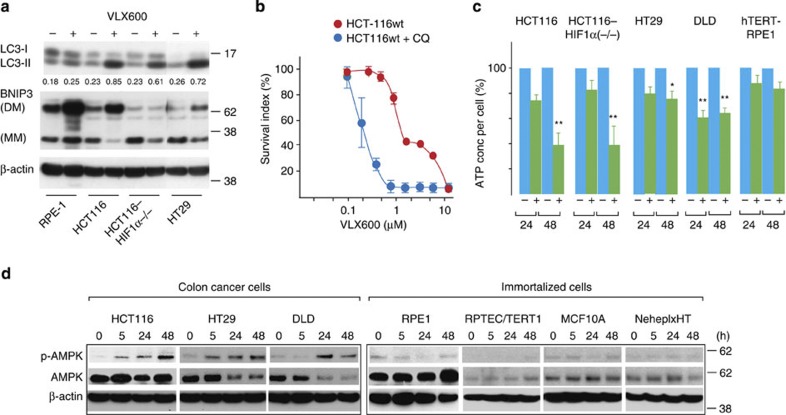
VLX600-induced autophagy is a protective response. (**a**) Analysis of LC3 and BNIP3 expression after VLX600 treatment of different human cell lines. Cells were treated with VLX600 (6 μM) for 24 h and analysed by western blotting. BNIP forms monomers (MM) and dimers (DM; stable in SDS). All lanes are from the same exposure of the same film. (**b**) Effect of chloroquine (CQ) on cytotoxicity of VLX600 in HCT116 monolayer cells. Cells were exposed to VLX600 in the presence of absence of 25 μM CQ and viability was determined after 72 h using the fluorometric microculture cytotoxicity assay. Shown are means±s.e.m. (*n*=3). (**c**) Levels of ATP in colon cancer cell lines and immortalized hTERT-RPE1 cells after different times of VLX600 exposure. The levels of ATP were corrected for the number of viable cells. Shown are means±s.e.m. (*P<0.05, **P<0.01; *t*-test; *n*=3). (**d**) Monolayer colon carcinoma and immortalized cells were exposed to 6 μM VLX600 and analysed for phospho-AMPK, AMPK and β-actin by western blotting at the times indicated. Note the induction of phospho-AMPK in colon cancer cells, but not immortalized cells.

LC3-II levels were strongly induced by VLX600 in HCT116, HCT116^HIF-1α−/−^ and HT29 cells, all sensitive to VLX600 (Fig. 4a). In contrast, LC3-II was only weakly induced in the relatively insensitive, untransformed hTERT-RPE1 cell line (Fig. 4a). These findings are consistent with induction of autophagy being a protective response to VLX600. The cytotoxic effect of VLX600 was indeed found to be potentiated by different autophagy inhibitors (Fig. 4b; Supplementary Fig. 4a,b).

We considered the possibility that autophagy was mediated via HIF-1α and BNIP3. BNIP3 is an HIF-1α-induced protein described to induce cytoplasmic vacuolization and autophagy^31^. BNIP3 was indeed induced by VLX600 in HCT116 cells, but not in HCT116^HIF-1α−/−^ cells (Fig. 4a). Since LC3-II induction was observed in the HIF-1α-defective cells (Fig. 4a), autophagy induction by VLX600 does not require BNIP3.

We hypothesized that the protective autophagy response is secondary to a bioenergetic catastrophe. We indeed observed HIF-1α-independent decreases in cellular ATP content in HCT116 cells after 24 and 48 h of drug exposure (Fig. 4c). Decreases in ATP were also observed in DLD and HT29 colon cancer cells, whereas untransformed hTERT-RPE1 cells did not show significant decreases (Fig. 4c).

AMP-activated protein kinase (AMPK) is phosphorylated in response to increasing AMP/ATP ratios in cells^32^, resulting in increased autophagy via mTOR signalling^33^. We examined AMPK phosphorylation in three colon cancer and four immortalized cell lines. Interestingly, AMPK was phosphorylated in all three colon cancer cell lines, with phosphorylated AMPK already detectable after 5 h drug exposure in HCT116 and HT29 cells (Fig. 4d; Supplementary Fig. 4c). In contrast, increases in AMPK phosphorylation were not observed in any of the immortalized cells (Fig. 4d). Taken together, our data suggest that VLX600 preferentially leads to decreased ATP levels in cancer but not normal cells.

### Mitochondrial dysfunction leads to larger glucose dependence

**Figure 5:**
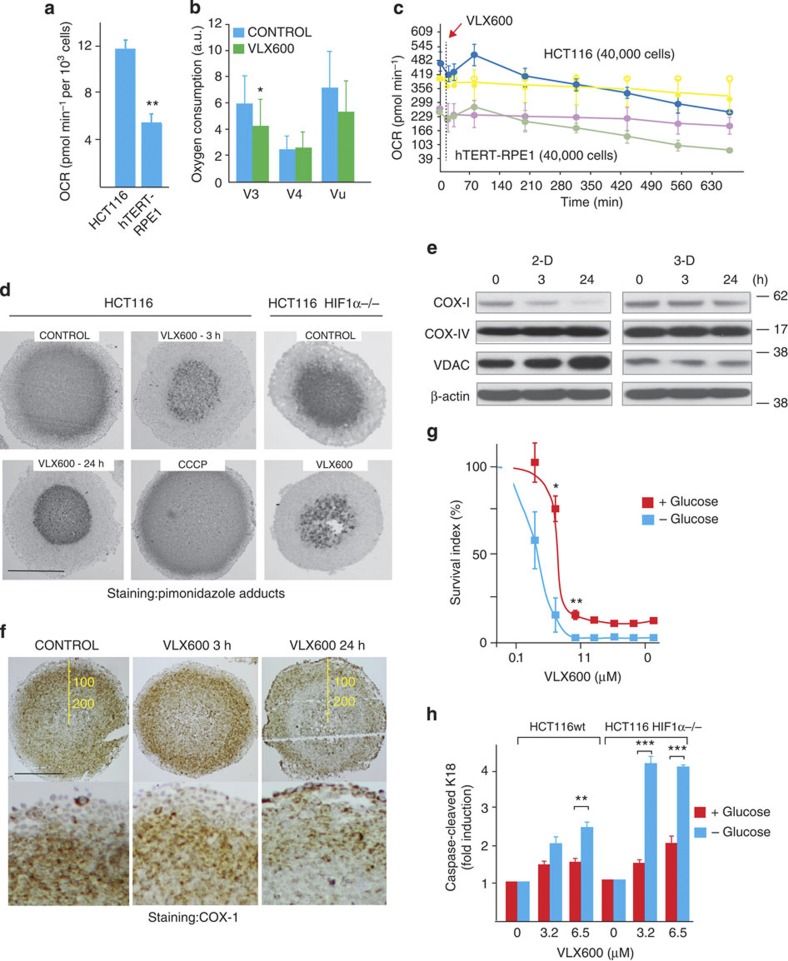
VLX600 causes mitochondrial dysfunction. (**a**) OCRs in HCT116 and immortalized hTERT-RPE1 cells in monolayer culture. OCR was determined in a Seahorse XF analyser and data represent means (± s.e.m.); **P<0.01 (*t*-test; *n*=3). (**b**) VLX600 inhibits oxygen consumption in HCT116 cells. State 4 respiration (V_4_) was measured in the presence of 1 μg ml^−1^ oligomycin, which blocks mitochondrial ATP synthase. Uncoupled respiration (V_u_) was assessed in the presence of 3 μM carbonyl cyanide *m*-chlorophenylhydrazone (CCCP). Experiments performed using a Clark-type electrode (*n*=3; means±s.e.m; *P<0.05, *t*-test). For the definition of state 3 and 4 respiration, see Chance and Williams^63^ (**c**) Transient increases followed by decreases in OCR following VLX600 exposure. OCR was determined using a Seahorse XF analyser. An ~50% decrease in OCR was observed over 10 h. Shown are means±s.d. (*n*=3). (**d**) VLX600 reduces MCS hypoxia. Spheroids (HCT116 or HCT116^HIF-1α−/−^) were exposed to vehicle, 6 μM VLX600 or the uncoupler CCCP, treated with pimonidazole, sectioned and stained with an antibody to pimonidazole adducts. Scale bar, 250 μm. (**e**) Effect of VLX600 on the expression of mitochondrial proteins. HCT116 cells grown in monolayer or as MCS were analysed for the expression of COX subunit 1 (COX-I), COX subunit-IV (COX-IV and VDAC. Cells were exposed to 6 μM VLX600 for the times indicated and analysed by immunoblotting. (**f**) Staining of sectioned HCT116 MCS for COX-1. Cells were exposed to 6 μM VLX600 for the times indicated, sectioned and stained. Scale bar, 250 μm. (**g**) HCT116 monolayer cells were exposed to different concentrations of VLX600 in glucose-containing or glucose-free medium. Cell viability was determined after 72 h using the fluorometric microculture cytotoxicity assay. Shown are means±s.e.m; *P<0.05 **P<0.01, *t*-test (*n*=3). (**h**) HCT116 or HCT116^HIF-1α−/−^ cells were exposed to VLX600 in the presence of absence of glucose as in **g**. The levels of caspase-cleaved K18 was determined after 24 h by ELISA. Shown are means±s.d.; **P<0.01, ***P<0.001, *t*-test (*n*=3).

We next sought to determine the nature of the metabolic deficiency elicited by VLX600. A clue to the nature of the deficiency came from the analysis of mitochondrial ΔΨ in treated cells, showing mitochondrial hyperpolarization in viable cells at 24 h (Supplementary Fig. 1k). Examination of mitochondrial function in HCT116 cells showed a high oxygen consumption rate (OCR) compared with hTERT-RPE1 cells (Fig. 5a), which was linked to ATP production (Supplementary Fig. 5a). VLX600 was found to induce significant decreases in V_3_ respiration after 6 h of exposure (Fig. 5b). Decreased OCRs, after a transient increase, were also observed in experiments where cellular oxygen consumption was analysed using a Seahorse extracellular flux analyzer (Fig. 5c). To examine whether VLX600 elicited a decreased oxygen consumption also in MCS, tissue oxygen tension was visualized by pimonidazole staining^34^. VLX600 exposure reduced the pimonidazole hypoxic fraction (pHF) of MCS to ~50% of control (from 63±13% to 32±4% at 3 h; *P*<0.01 (mean±s.d.; *t*-test); Fig. 5d). The effect was observed after 3 h of drug exposure and persisted until 24 h (Fig. 5d). A control experiment showed that increases of oxygen consumption elicited by the mitochondrial uncoupler carbonyl cyanide *m*-chlorophenyl hydrazone lead to an increased spheroid pHF (Fig. 5d). MCS formed from HCT116^HIF-1α−/−^ cells, previously shown to consume comparatively low levels of oxygen^35^, showed a smaller pHF that was further decreased by VLX600 (Fig. 5d).

We examined the effects of VLX600 on the expression of mitochondrial proteins in HCT116 monolayer and MCS cultures (Fig. 5e). Cytochrome *c* oxidase (COX; complex IV) is the last enzyme complex in the mitochondrial electron transport chain and the primary site of cellular oxygen consumption^36^. COX-1 (subunit 1) expression decreased in HCT116 cells exposed to VLX600, particularly in monolayer cultures (Fig. 5e). COX-IV expression was not altered, whereas voltage-dependent anion channel (VDAC) expression increased in monolayer cultures but not in MCS (Fig. 5e). We examined COX-1 by immunohistochemistry (Fig. 5f). Staining was not uniform in MCS with the strongest staining observed in layers 50–150 μm below the MCS surface and also observed at deeper layers (Fig. 5f). The COX-1 staining pattern was altered by VLX600 treatment with weak staining observed in the deeper areas (>100 μm from the surface) 24 h post drug treatment. Alterations in COX-1 were not observed at 3 h, however, a time point when decreased pimonidazole staining was already evident (Fig. 5d,f). We conclude that the expression of COX-1, essential for mitochondrial OXPHOS, decreases after exposure of monolayer cells to VLX600 and is also decreased in MCS core regions.

Cells in core areas of MCS lack glucose supplies and are predicted to be particularly sensitive to mitochondrial dysfunction. We directly tested whether glucose starvation increases the sensitivity of HCT116 cells to VLX600. Indeed, exposure to VLX600 in glucose-free medium resulted in increased cytotoxicity and apoptosis (Fig. 5g,h). Glucose-deprived HCT116^HIF-1α−/−^ cells were particularly sensitive to VLX600-induced apoptosis (Fig. 5h).

### Characterization of mitochondrial dysfunction

**Figure 6:**
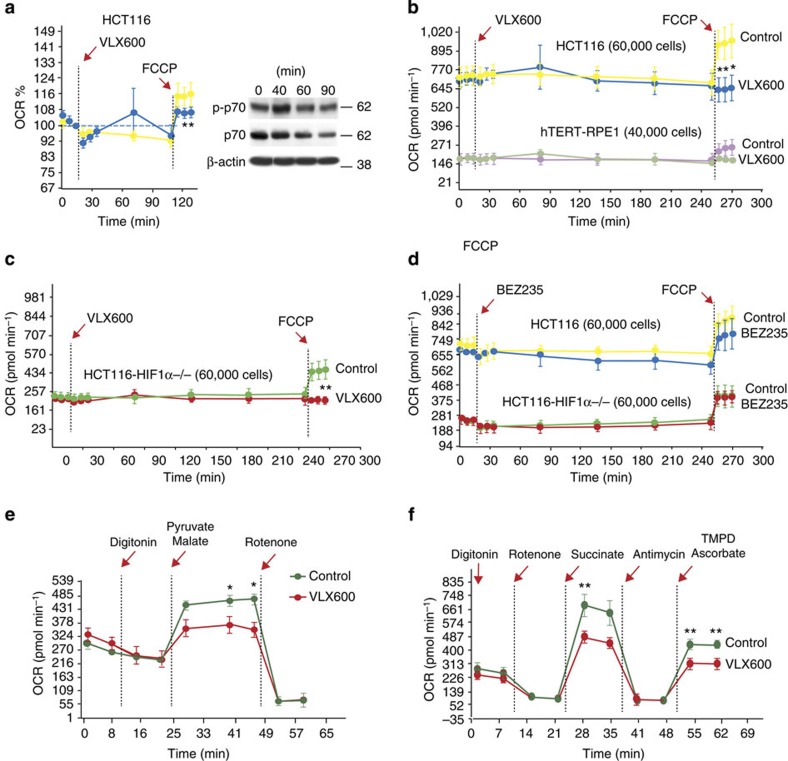
Characterization of mitochondrial dysfunction. (**a**) Impaired stimulation of OCR by carbonyl cyanide-4-trifluorometh-oxyphenylhydrazone (FCCP) after 90 min of VLX600 exposure. OCR was analysed using a Seahorse XF analyser. VLX600 (6 μM) was injected after 18 min and OCR was recorded. FCCP was injected where indicated. Phosphorylation of p70 was examined by western blotting during the same time period. Shown are means±s.d. (*n*=3); *P<0.05 (*t*-test). (**b**) Loss of stimulation of OCR by FCCP after 4 h of exposure of HCT116 or hTERT-RPE1 cells to VLX600 (6 μM). Shown are means±s.d. (*n*=3), *P<0.05 (*t*-test). (**c**) Loss of stimulation of OCR by FCCP after 4 h of VLX600 exposure of HCT116^HIF-1α−/−^ cells. Shown are means±s.d. (*n*=3), **P<0.01 (*t*-test), (**d**) NVP-BEZ235 does not abolish mitochondrial reserve capacity for oxygen consumption after FCCP treatment. Cells were exposed as in **a**–**c** except that NVP-BEZ235 was used (0.2 μM). Shown are means±s.d. (*n*=3). (**e**) VLX600 partially inhibits complex I. Pyruvate and malate were added to digitonin-permebilized cells and OCR was monitored. Shown are means±s.d. (*n*=3), *P<0.05 (*t*-test). (**f**) VLX600 inhibits complex II and IV. Succinate and *N*,*N*,*N*’,*N*’-tetramethyl-1,4-phenylene diamine/ascorbate were added to digitonin-permeabilized cells and OCR was monitored. Rotenone was added before succinate to inhibit complex I. Shown are means±s.d. (*n*=3), **P<0.01 (*t*-test).

We further characterized the mitochondrial dysfunction induced by VLX600. We added the uncoupler carbonyl cyanide-4-trifluorometh-oxyphenylhydrazone to HCT116 cells at different times of VLX600 exposure and measured OCR. Already after 90 min, uncoupled respiration was impaired (Fig. 6a), and no stimulation by the uncoupler was observed after 4 h (Fig. 6b). Importantly, no effects on p70 phosphorylation were observed at 90 min, showing that the effect on mitochondria preceded mTOR inhibition (Fig. 6a). The inhibition of uncoupled respiration was also observed in HCT116^HIF-1α−/−^ cells (Fig. 6c) and in hTERT-RPE1 cells (Fig. 6b). The PI3KCA/mTOR inhibitor NVP-BEZ235 decreased oxygen consumption during 4 h incubation, but did not affect mitochondrial reserve capacity (Fig. 6d). Using digitonin-permeabilized HCT116 cells, we examined the utilization of different mitochondrial substrates in VLX600-exposed cells. We found partial inhibition of complex I, II and IV (Fig. 6e,f). We conclude from these results that VLX600 has profound effects on mitochondrial OXPHOS by inhibiting the function of complexes in the electron transport chain.

Mitochondrial dysfunction may be expected to result in generation of reactive oxygen species, which are cytotoxic to tumour cells^37^. We found, however, that VLX600 in fact reduced reactive oxygen species production to ~50% of that of control cells (Supplementary Fig. 6), possibly because of the decreased rate of respiration.

### Antitumour activity of VLX600 in human tumour xenografts

**Figure 7:**
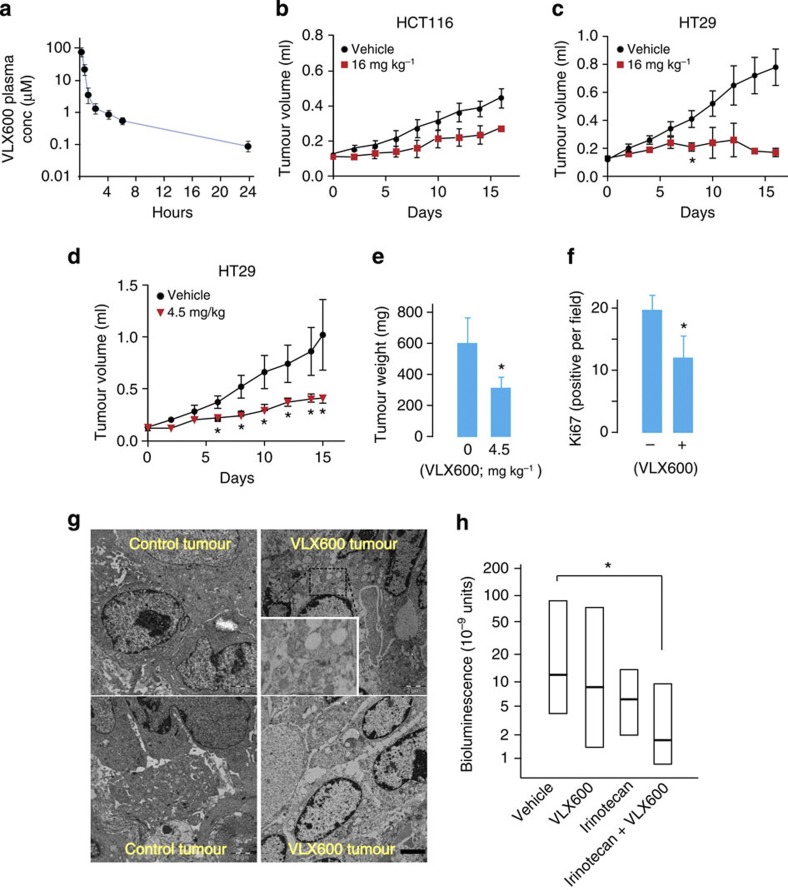
*In vivo* activity of VLX600 on colon carcinoma xenografts. (**a**) Pharmacokinetic profile of VLX600 following intravenous injection (16 mg kg^−1^). Plasma was analysed by mass spectrometry (*n*=3; means±s.e.m.). (**b**) Antitumour effect of VLX600 on HCT116 colon carcinoma xenografts (16 mg kg^−1^). Mice were treated every third day for 16 days (means±s.d.; *n*=7). (**c**) Antitumour effect of VLX600 on HT29 xenografts (16 mg kg^−1^). Mice were treated with drug intravenously every third day for 16 days (*n*=8 for control; *n*=11 for VLX600). The study was only carried through for two animals from the VLX600 group because of toxicity at the injection site (tail vein); *P*=0.02 (*t*-test) for six animals in VLX600 group at day 8. Shown are means±s.d. (**d**) Antitumour effect of VLX600 on HT29 xenografts. Mice were treated with drug every third day for 15 days (*n*=6 in vehicle; *n*=7 in 4.5 mg kg^−1^ VLX600 group; all animals from treated groups completed the study). Differences between treated groups and control are significant at *P*<0.05 (*t*-test) from day 6. Shown are means±s.d. (**e**) Animals were killed, and the tumours were excised and weighed (means±s.d.; *P*<0.05 (*t*-test)). Same animals as in **d**. (**f**) Tumour cell proliferation in VLX600-treated tumours. Animals were treated with 16 mg kg^−1^ VLX600 24 h before killing. Tumours were sectioned and stained for Ki67. Labelling index was scored blindly (*n*=3; shown are means±s.d.; * *P*<0.05 (*t*-test)). (**g**) Electron micrographs of xenografted HCT116 tumours from mice repeatedly treated with VLX600 intravenously (last treatment 24 h before the killing) and control tumours. Viable cells contained multiple vesicles and few discernible mitochondria (scale bar, 2 μm). (**h**) HT29-LUC colon carcinoma cells were implanted orthotopically in SCID mice (*n*=9 or 10). Animals were treated with irinotecan (45 mg kg^−1^, days 11 and 15) and/or VLX600 (8 mg kg^−1^, every other day × 5, days 11–20) and bioluminescence was measured at day 35. Owing to the large variation between animals, medians and quartiles are shown (the Mann–Whitney test, *P<0.05).

To study the effects of VLX600 *in vivo*, the drug was injected intravenously in NMRI mice. At the maximally tolerated dose of 16 mg kg^−1^, an initial plasma concentration of ~100 μM was observed (Fig. 7a), >10-fold the IC_50_ of tumour cell lines and primary patient colorectal cancer cells *in vitro*. The compound was rapidly distributed and finally eliminated with a half-life of ~4–5 h. Using the maximally tolerated dose, antitumour activity was observed in both HCT116 and HT29 colon cancer xenografts (Fig. 7b–e). Importantly, minimal systemic toxicity was observed as evidenced by no loss of body mass and no or minor changes in plasma parameters such as liver alanine aminotransferase, blood glucose and total protein (Supplementary Fig. 7a,b).

We examined sections from VLX600-treated HCT116 tumours. VLX600 treatment resulted in a decreased Ki67-labelling index (Fig. 7f) consistent with growth arrest. Large cytoplasmic vesicles were also observed (Fig. 7g), suggesting that the compound induced the formation of autolysosomes also *in vivo*.

We examined VLX600 in combination with drugs routinely used for clinical management of colon carcinoma patients. Synergy was observed with both irinotecan and oxaliplatin, whereas VLX600 and 5-fluorouracil showed additive effects (Supplementary Fig. 7c). The combination of VLX600 and irinotecan was tested in an orthotopic colon cancer model using a relatively moderate treatment schedule for each drug, which did not significantly reduce tumour growth as single treatments (VLX600: five cycles 8 mg kg^−1^; irinotecan: two cycles 45 mg kg^−1^). The median bioluminescent signal from animals treated with the combination was ~10% of vehicle controls (Fig. 7h; *P*<0.05 (Mann–Whitney)), suggesting that the combination of VLX600 with irinotecan was effective in inhibiting tumour growth.

**Figure 8:**
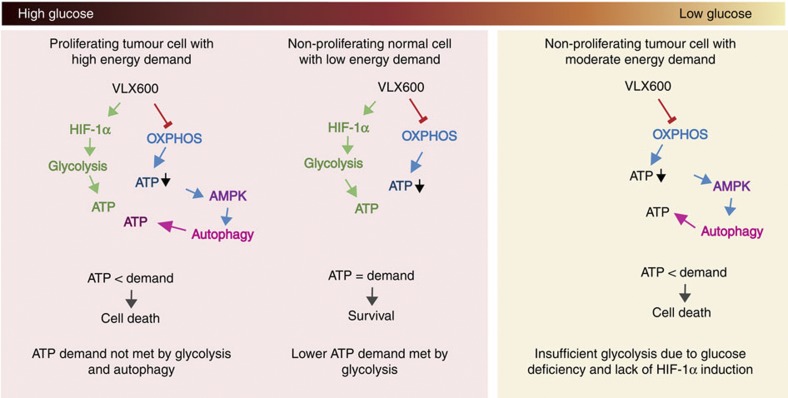
Model for the cytotoxic effects of VLX600 on tumour cells. VLX600 impairs OXPHOS and induces a HIF-1α-dependent shift to glycolysis. This shift will be protective to tumour cells and normal cells in microenvironments where glucose is available. Tumour cells will not meet their energy demands solely by glycolysis, leading to induction of autophagy. In metabolically compromised microenvironments, the ability to shift to glycolysis will be limited because of glucose unavailability and lack of HIF-1α-stabilization. The lack of metabolic plasticity in the deep tumour parenchyme represents a therapeutic opportunity.

On the basis of the results of this study, a model for the effect of VLX600 on normal and tumour cells in different microenvironments is presented in Fig. 8.

## Discussion

Screening and validation experiments identified the small molecule VLX600 to have the unusual ability to reduce the viability of quiescent cells in a 3-D *in vitro* tumour model. VLX600 did not kill non-proliferating immortalized cells and did not induce detectable systemic toxicity in mice. The effect on colon cancer tumour cells obtained directly from patients demonstrated sensitivity within the plasma concentration range achievable *in vivo* and activity was observed against colon cancer models *in vivo*. Furthermore, VLX600 was found to increase the efficacy of irinotecan, a drug used to treat colon carcinoma patients. VLX600 is therefore a candidate drug for clinical trials.

VLX600 was demonstrated to induce mitochondrial dysfunction, associated with a dramatic decrease in spheroid hypoxia. The decrease in mitochondrial OXPHOS will necessitate a shift to other modes of energy generation for maintenance of cellular ATP levels. However, a shift to glycolysis will be hampered by the low glucose availability in MCS^21^,^22^ and in solid tumours^15^,^19^,^20^. The low glucose content in solid tumour tissues is believed to result both from poor blood supply and from high glucose consumption by cancer cells^15^. Tumour cells in metabolically compromised areas will depend on both glycolysis and OXPHOS for energy production, and the resulting limited plasticity is likely to represent a therapeutic opportunity.

VLX600 induced a strong shift to glycolysis that will be protective to tumour cells in microenvironments where glucose is available, and may also be protective to cells in healthy and vascularized tissues. The shift to glycolysis was found to be dependent on HIF-1α and we found that disruption of HIF-1α indeed resulted in an increased sensitivity to the drug. Interestingly, since HIF-1α was not induced in MCS core regions, possibly owing to lack of stabilization of HIF-1α under low glucose conditions^26^, cells in these microenvironments will be less protected. This lack of HIF-1α stabilization will further decrease the ability of cells in core regions to adjust to drug-induced decreases in OXPHOS. Our findings are summarized in the model shown in Fig. 8.

Metabolomic studies have shown that malignant tumours have elevated metabolic turnover rates and thus higher demands in energy supply^19^,^20^,^38^. It was also recently reported that Li–Fraumeni syndrome family members who carry germline mutations in TP53 show increased OXPHOS of skeletal muscle^39^. Increased energy demands cannot be met by solely glycolysis, and recent data have indeed showed hyperactivation of oxidative mitochondrial metabolism in epithelial cancer cells^40^. Indeed, cancer cells are dependent on mitochondrial function for growth and tumorigenicity^41^,^42^, and increased mitochondrial biogenesis promotes breast cancer tumour growth^43^. We found an ~2.5-fold higher OCR in HCT116 colon cancer cells compared with non-transformed hTERT-RPE1 cells. HCT116 cells therefore appear to be highly dependent on OXPHOS for energy production also at conditions of high glucose availability. Consistent with this hypothesis, VLX600 was found to induce decreases in ATP and increases in AMPK phosphorylation in HCT116 cells and two additional colon cancer cell lines. In contrast, AMPK phosphorylation was not induced in any of the four immortalized cell lines examined. This difference in the response to VLX600 between cancer cells and normal cells is likely to be important for the selective cytotoxicity of the compound.

Mitochondrial inhibitors have been reported to possess antitumour activity^44^,^45^,^46^. Examples are the mitochondrial dye Rho123 (Bernal *et al*.^44^), which inhibits mitochondrial OXPHOS^47^, the complex V inhibitor efrapeptin F^46^ and mitochondria-targeted lipophilic cations^48^. Metformin is a commonly prescribed anti-diabetic drug that inhibits mitochondrial complex I (Owen *et al*.^49^) and increases cellular glucose uptake^50^. Metformin has been shown to inhibit tumour formation in diabetic patients^51^ and in mouse animal models^52^,^53^. The antitumour efficacy of metformin is likely to be limited by the hydrophilic properties of this drug (XlogP, −1.3), restricting penetration into the deep tumour parenchyme^54^. VLX600 is a lipophilic cation (XlogP, 2.85; estimated pKa, 3.5). These properties will facilitate penetration into cells and accumulation in the mitochondria.

In conclusion, our screen for compounds that decrease the viability of quiescent cells in 3-D tumour spheroids resulted in the identification of an inhibitor of mitochondrial OXPHOS. Our findings suggest that tumour cells in nutritionally compromised microenvironments are sensitive to disturbances of mitochondrial function, resulting in a bioenergetic catastrophe. Since conventional cytotoxic anticancer agents are significantly less effective under conditions of nutrient depletion^55^, this type of mechanism will be interesting to exploit for therapy. Our finding showing enhancement of the antitumour effect of irinotecan by VLX600 is promising and VLX600 is currently in development for a phase 1 clinical trial in patients with solid tumours.

## Methods

### Reagents

Antibody to p62 (# 610833) was purchased from BD Biosciences (Franklin Lakes, NJ, USA). Antibody to BNIP3 (# 10433) and COX (subunit 1; # 90668) were from Abcam (Cambridge, MA, USA). Antibody to β-actin (# A5316) was from Sigma-Aldrich (St Louis, MO, USA). Antibodies to 4EBP1 (#9452), phospho-4EBP1 (# 9459), p70-S6K (# 9202), phospho-p70-S6K (# 9205), AMPK (# 2532), phospho-AMPK (# 4188), calnexin (#C5C9), COX (subunit 4) (#4844), VDAC (# 4661), Bip/Grp78 (# 3177) and LC3 (# 2775) were from Cell Signaling (Danvers, MA, USA). Antibody to p27/Kip1 (# 610241) was from BD Transduction Laboratories (San Jose, CA). Antibody to HIF-1α was from Novus Biologicals (# NB100-105). Antibody to eIF2-α (# sc-11386) and phospho-eIF2-α (# sc-12412) were from Santa Cruz Biotechnology (Santa Cruz, CA). The kit for pimonidazole staining was from NPI Inc. (Belmont, MA). ATP assay kit was from Promega (Madison, WI, USA). The DiverseSet (10,000 compounds) used for screening was purchased from ChemBridge. The original ChemBridge compound 5625138 was resynthesized in gram scale by OncoTargting AB (Uppsala, Sweden). The resynthesized compound generated identical results as the original compound. All compounds were dissolved in dimethylsulphoxide (DMSO). A final concentration of 0.5% DMSO was reached in cell cultures; control wells received solvent only (that is, final 0.5% DMSO concentration).

### Cell culture

The human colon carcinoma cell lines HCT116, HT29, SW620, HT8, DLD and RKO were obtained from the American Type Culture Collection, Manassas, VA, USA). The normal epithelial cell line hTERT-RPE1 was obtained from Clontech (Palo Alto, CA), and cell lines MCF10A (normal epithelial), RPTEC/TERT1 (normal renal) and NeHepLxHT (normal hepatic) were obtained from American Type Culture Collection. All experiments on purchased cell lines were performed with less than 6 months passages in our laboratory after receipt or resuscitation. All cell lines were sub-cultivated and grown in supplemented medium as recommended by the providers. The HCT116 colon carcinoma derivatives HCT116 horseradish peroxidase (HRP) enhanced green fluorescent protein (kindly provided by Dr Monti), HCT116^p53−/−^ (kindly provided Dr B. Vogelstein ) and HCT116^HIF-1−/−^ cell lines have been described previously^35^,^56^,^57^. HCT116 colon carcinoma cells were maintained in McCoy’s 5A modified medium/10% fetal calf serum at 37 °C in 5% CO_2_. Viability measurement were performed using an acoustic Echo liquid handler (Labcyte) and the fluorometric microculture cytotoxicity assay as a read-out^58^. Tumour samples were obtained from 22 patients with colorectal cancer peritoneal carcinomatosis. The patient sampling was approved by the local ethics committee at the Uppsala University hospital.

### Generation of spheroids and screening

Spheroids were prepared using a modification of our previously described method^17^. A cell suspension containing 10,000 cells (200 μl) was added to each well of poly-HEMA-coated 96-well plates. Wells were overfilled by adding 170 μl media to acquire a convex surface curvature and plates were inverted to allow cells to sediment to the liquid–air interface. Plates were returned to normal after 24 h incubation, excess media were removed by aspiration and incubated for 4 days before drug exposure. Screening was performed by exposing spheroids to drug for 6 h, followed by washing and incubation for an additional 66 h. Each well contained 200 μl medium and received 1 μl drug solution (drugs were dissolved in 100% DMSO). Control wells received 1 μl dimethylsulphoxide. At the end of incubation, viability was determined using the acid phosphatase method^59^. After washing twice with PBS buffer, spheroids were lysed in 100 μl of 0.1 M sodium acetate, 0,1% Triton X-100, *p*-nitrophenyl phosphate (Pierce Biotechnology Inc, Rockford, IL) and incubated for 90 min at 37 °C. At the end of the incubation, 10 μl NaOH was added to each well and A405 nm determined. Background activity was subtracted.

### Western blotting

Cell extract proteins were resolved by Tris-Acetate PAGE gels (Invitrogen, Carlsbad, CA) and transferred onto polyvinylidene difluoride membranes, which were incubated overnight to antibodies, washed and incubated with HRP-conjugated anti-rabbit Ig (Amersham Biosciences, Little Chalfont, UK) for 1 h. Antibodies were used at the following dilutions: HIF-1α (1:200), β-actin (1:10,000), LC3 (1:1,000), BNIP3 (1:500), AMPK (1:1,000), phospho-AMPK (1:2,000), COX-1 (1:1,000), COX-IV (1:1,000), VDAC (1:1,000), p70 (1:1,000), phospho-p70 (1:1,000), eIF2-α (1:200), phospho-eIF2-α (1:200), caspase-4 (1:1,000), 4EBP1 (1:1,000) phospho-4EBP1 (1:1,000) and p62 (1:400). Peroxidase activity was developed by SuperSignal West Pico (Pierce Biotechnology). Unedited versions of Fig. 3d (LC3) and Fig. 4a (LC3 and BNIP3) are shown in Supplementary Fig. 8.

### Chemical synthesis and analytical procedures

Chemical synthesis of VLX600 was outsourced to OncoTargeting AB (Uppsala, Sweden) and Niels Clauson-Kaas A/S (Farum, Denmark). In brief, the synthesis was performed in three steps starting with commercially available 7-methylisatin and thiosemicarbazide. The formed cyclized 1,2,4-triazino-3-thione derivate was further reacted with hydrazine monohydrate, yielding a hydrazine intermediate, which was finally reacted with 2-acetylpyridine to yield VLX600 as a free base (purity >99%). Determination of VLX600 in the plasma was performed using an Agilent 6110 single quadrupole mass spectrometer.

### Treatment of mouse xenografts

For subcutaneous models, a 100-μl cell suspension containing 5 × 10^6^ HT29 or HCT116 cells was injected subcutaneously at the right rear flank of female NMRI *nu/nu* mice (8–10 weeks old). When tumours had grown to a size of 0.1 ml, mice were randomized into control or treatment groups and injected with VLX600 intravenously (as specified in the legends) and tumour size was measured. VLX600 was dissolved in 4.73 mM HCl to a concentration of 0.6 mg ml^−1^ and diluted with 0.9% NaCl. Some tumours were fixed with 2.5% glutaraldehyde at +4 °C overnight. The piece was excised from the tumour to make sure that the whole tumour was represented (a wedge-shaped slice from the centre to the outer surface). The tissues were post-fixed in 1% osmiumtetraoxide (OsO_4_), dehydrated and embedded in epon (epoxy resin). Ultrathin sections were prepared for analysis in a transmission electron microscope. Plasma samples were analysed for alanine aminotransferase and other analytes using an Abaxis VetScan VS2 instrument. Subcutaneous models were performed by Adlego AB, Stockholm (with the approval of Stockholm North Ethics committee). For determination of orthotopic topic tumour growth, HT29-LUC human colon tumour cells (5 × 10^6^ in a volume of 50 μl) were injected into the intestinal caecal wall of male athymic nude mice. Nine to ten animals were used in each group. Eleven days after tumour implantation, tumour-bearing animals were size rank-matched into five treatment groups and treatments were started. VLX600 (8 mg kg^−1^) was injected five times (every second day), and irinotecan (45 mg kg^−1^) was injected two times (4 days apart) starting on day 11. For imaging, mice were injected intraperitoneally with 150 mg kg^−1^ D-luciferin (Promega) followed by anesthetization in 2–3% isoflurane atmosphere. After 10–12 min of biodistribution time, mice were imaged using a charge-coupled device camera (Xenogen IVIS Lumina System) to evaluate the bioluminescence in the animal. Photon emission was measured as whole-body radiance and the individual regions of interest were manually selected. Data were expressed as photon s^−1^ cm^−^^2^ per sr. Imaging data were not normally distributed and data were treated using non-parametric statistics. These experiments were performed by Accelera (Neviano, Italy) with a permit from the Nerviano Medical Sciences (NMS) Ethic Committee.

### Determination of apoptosis by ELISA

Following drug exposure, NP40 was added to the culture medium to 0.1% to extract caspase-cleaved K18 from spheroids and to include material released to the medium from dead cells. Caspase-cleaved keratin-18 (K18-Asp396) was determined using 25 μl medium/extract using the M30 CytoDeath enzyme-linked immunosorbent assay (ELISA) (a variant of the M30-Apoptosense ELISA^60^ developed for *in vitro* use (Peviva AB, Bromma, Sweden).

### Patient samples

Tumour samples were obtained from 22 patients with colorectal cancer peritoneal carcinomatosis. All except two samples were from patients previously treated with chemotherapy. The tumour sampling was approved by the Regional Ethical Board at the Uppsala University Hospital and all patients provided written informed consent. The tumour tissue obtained at the cytoreductive surgery was minced into small pieces and tumour cells were then isolated by collagenase dispersion followed by Percoll (Pharmacia Biotech, Uppsala, Sweden) density gradient centrifugation. Cell viability was routinely >90% as determined by trypan blue exclusion test and the proportion of tumour cells in the preparations as judged by inspection of May–Grünwald–Giemsa-stained cytospin preparations was >70%. Cell culture medium RPMI 1640 supplemented with antibiotics was used throughout. Three hundred and eighty-four-well microplates (Nunc, Roskilde, Denmark) were prepared with 5 μl VLX600 per well at 10 times the final drug concentration. The plates were then stored at −70 °C until further use. Tumour cells (5,000 cells per well in duplicates) were seeded in the drug-prepared 384-well plates. Three columns without drugs served as controls and one column with medium only served as blank. The plates were incubated at 37 °C for 72 h and cell viability was then analysed by measurement of fluorescence from viable cells after 40 min incubation with fluorescein diacetate. The method is based on measurement of fluorescence generated from the hydrolysis of fluorescein diacetate to fluorescein by cells with intact plasma membranes. Cell survival, expressed as survival index is defined as fluorescence in test wells divided by fluorescence of control wells, with blank values subtracted, multiplied by 100.

### Fluorescence-based HIF-1 analysis

HCT116 HRP enhanced green fluorescent protein cells were plated (10,000 cells per well) on 96-well plates (Nunc) and cultured in complete medium. Cells were treated with VLX600 and fluorescence was determined after 24 h using an automated IncuCyte-FLR 20X phase contrast/fluorescence microscope (Essen Instruments, Ann Arbor, MI). Average object summed intensity was calculated (triplicate wells, four images per well) using the IncuCyte software (Essen Instruments).

### High content analysis

Multiparametric high content evaluation of oxidative stress and LC3 was performed with dedicated kits (Thermo Fisher Scientific) in HCT116 cells using Cellomics ArrayScan VTI HCS Reader (oxidative stress 1, catalogue # 8401002; LC3B acidic vacuoles, catalogue # 8407802) according to the manufacturer’s instructions. For these assays, cells were seeded into 96-well plates (PerkinElmer Inc., Wellesley, MA, USA), left to attach overnight, before test compounds were added. Oxidative stress was studied using the Oxidative Stress I Kit containing dihydroethidium probe and Hoechst 33342. HCT116 cells were exposed to VLX600 and the positive control rotenone (Sigma-Aldrich) for 24 h. Images were acquired in each fluorescence channel for at least 1,000 cells per well (× 20 objective). LC3B were analysed using × 20 objective, 24 and 42 h after addition of 6 μM VLX600. Acidic vacuoles in HCT116 cells were stained using Lyotracker Red DND-99 (Invitrogen, Molecular Probes Inc., OR, USA) after 42 h treatment with 6 μM VLX600 followed by wash and staining of nuclei with 10 μM Hoechst 33342 (Sigma-Aldrich) in fixation solution containing 3.7% formaldehyde (Sigma-Aldrich).

### Microarray gene expression analysis

In a first experiment, HCT116 cells were grown as spheroids and monolayer cultures, washed with PBS and total RNA was prepared using RNeasy miniprep kit (Qiagen, Chatsworth, CA). Starting from 2 μg of total RNA, gene expression analysis was performed using Genome U133 Plus 2.0 Arrays according to the GeneChip Expression Analysis Technical Manual (Rev. 5, Affymetrix Inc., Santa Clara, CA). Raw data were normalized using MAS5 (Affymetrix Inc.). Gene expression profiles were generated for two independent monolayer cultures and two independent spheroid cultures, and mean ratios for each probe were calculated and used to generate a ranked gene list. In a second experiment, HCT116 and HCT116^HIF-1−/−^ cells were grown as spheroids cultures. The cells were treated with 6 μM VLX600 for 6 h or left untreated (controls). Gene expression analysis was performed and ratios between treated and untreated cultures were established. Only probes with expression over 300 expression units and present calls were used in the GSEA^18^. Displayed *P* values refer to the nominal *P* values generated after 1,000 permutations. Raw and normalized gene expression data from the first and second experiment have been deposited at the Gene Expression Omnibus (http://www.ncbi.nlm.nih.gov/geo/) with series records GSE53631 and GSE53777, respectively.

### Immunological assays

MCS produced by the hanging drop method in 96-well plates were fixed in 2% buffered formalin, dehydrated, embedded in paraffin and sectioned. Each sample contained 24 spheroids (spheroids from each 96-well plate were pooled into four groups). The sections were deparaffinized with xylene, rehydrated and microwaved and then incubated overnight with the monoclonal primary antibodies diluted in 1% (wt/vol) BSA and visualized by standard avidin–biotin–peroxidase complex technique (Vector Laboratories, Burlingame, CA, USA). Counterstaining was performed with Mayer’s haematoxylin. Antibody MIB-1 (against the nuclear proliferation-associated antigen Ki67; Immunotech SA, Marseille, France) was used at a dilution 1:400; antibody against active caspase-3 (Biosite, BS7004) was used at 1:200; antibody to Bip/Grp78 (# 3177) was used at 1:200. HIF-1α immunohistochemistry was performed as described^61^.

### Lactate production assay

A total of 300,000 cells were plated in a six-well plate and exposed to VLX600 or NVP-BEZ235 next day for 24 h. Supernatant was collected and measured by lactate assay kit from BioVision (Mountain View, CA, USA) as per the described protocol.

### Glucose determination

Spheroids were trypsinized and washed in PBS. After washing, the cells were collected and diluted with 1 ml PBS. Cells were counted using a FACS Calibur instrument (Becton Dickinson, Mountain View, CA) using Cellquest software (Becton Dickinson). Then, cells were lysed by performing freeze-and-thaw method three times, and the supernatant was used for glucose concentration test using a glucose assay kit (Cambridge Science Park, Cambridge UK).

### ATP assay

In all, 10,000 cells were plated in each well in a 96-well plate and exposed to VLX600 for 24 or 48 h; ATP was measured by ATP assay kit from Promega.

### Measurements of oxygen consumption

Changes in the oxygen concentration were monitored with an oxygen electrode (Hansatech Instruments, Norfolk, UK) and analysed with the OxygraphPlus software (Hansatech Instruments) as described^62^. Cells were trypsinized, spun down and resuspended in 300 μl of the same medium. Cells were placed in respiratory chamber and basal respiration was monitored for 3–4 min. State 4 respiration was measured in the presence of 1 μg ml^−1^ oligomycin that blocks mitochondrial ATP synthase. Uncoupled respiration was assessed in the presence of 3 μM carbonyl cyanide *m*-chlorophenyl hydrazone. The Seahorse XF analyser was used as recommended by the manufacturer (Seahorse Bioscience, North Billerica, MA, USA). Cells were plated in 100 μl culture medium in XF24-well cell plates with blank control wells. Before the measurements, medium was replaced with 500 μl Seahorse assay media (1 mM pyruvate, 25 mM glucose) at 37 °C without CO_2_ for 1 h. OCR values were measured by XF24 extracellular flux analyzer. For analysis of electron flow, cells were permeabilized with digitonin (6 mg per 10^6^ cells) in 140 mM KCl, 5 mM KH_2_PO_4_, 1 mM MgCl_2_, 1 mM ADP and 5 mM Tris, pH 7.2 at 37 °C. Concentrations of substrates and inhibitors: malate, pyruvate and succinate all at 5 mM; rotenone 1 μm, malonate 10 mM, antimycin A 1 μM, ascorbate 5 mM and *N*,*N*,*N*’,*N*’-tetramethyl-1,4-phenylene diamine at 0.5 mM.

### Statistical analysis

Student’s *t*-test or Mann–Whitney tests were performed using Prism software for Apple computers. Statistical significance in *t*-test are plotted as following: *P<0.05, **P<0.01 and ***P<0.001.

## Author contributions

X.Z., M.F., E.H., W.F., M.H.O, V.G. and L.R. performed the experiments and performed data analysis; A.D.M., L.D., S.P., L.A.K.S., P.D., J.G., P.N., R.L. and S.L. performed data analysis. All authors contributed ideas, discussed the results and wrote the manuscript.

## Additional information

**How to cite this article:** Zhang, X. *et al*. Induction of mitochondrial dysfunction as a strategy for targeting tumour cells in metabolically compromised microenvironments. *Nat. Commun.* 5:3295 doi: 10.1038/ncomms4295 (2014).

**Accession codes**: The microarray data have been deposited in the Gene Expression Omnibus under accession codes GSE53631 and GSE53777.

## Supplementary Material

Supplementary Information Supplementary Figures 1-8
