# Supplementary material for: Induction of mitochondrial dysfunction as a strategy for targeting tumour cells in metabolically compromised microenvironments
Source: Nat Commun. 2014 Feb 18;5:3295. doi: 10.1038/ncomms4295 (PMC3929804; doi:10.1038/ncomms4295)
Supplement: Supplementary Information — Supplementary Figures 1-8 [file ncomms4295-s1.pdf]

SUPPLEMENTARY INFORMATION

Supplementary Figure 1

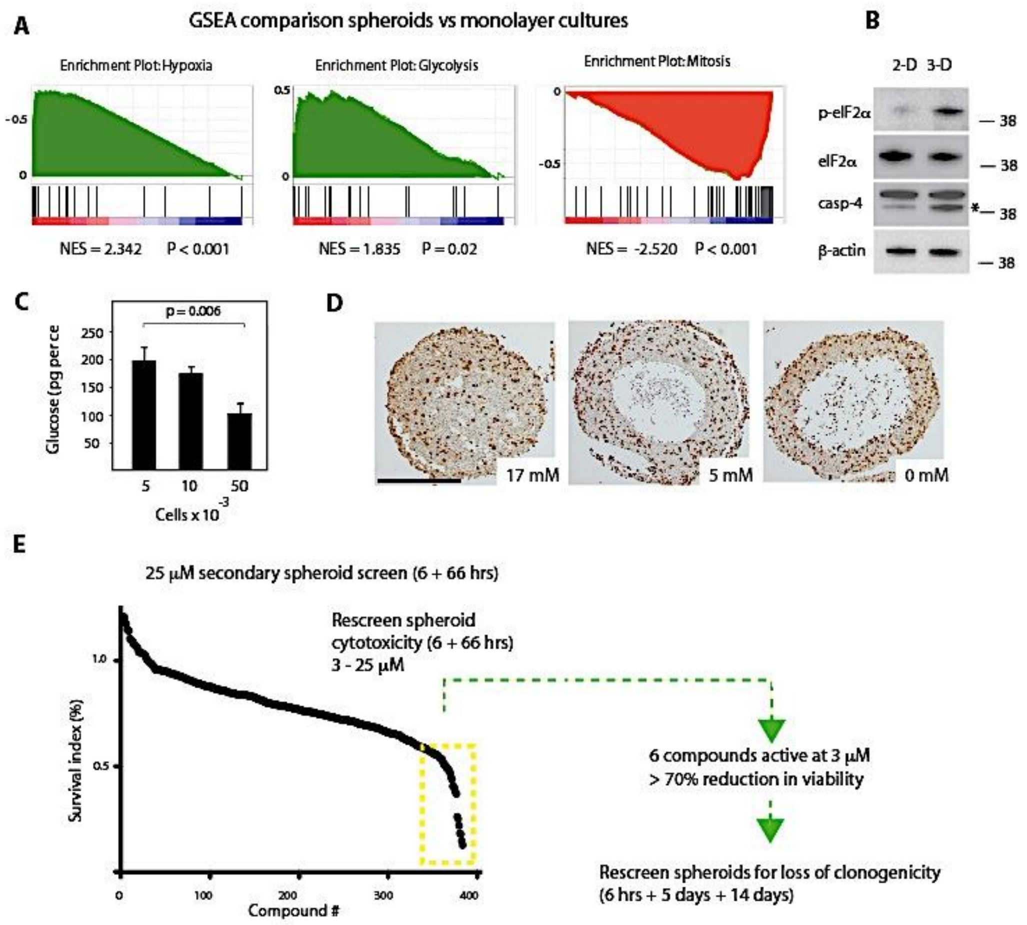

(a) Gene Set Enrichment Analysis (GSEA) plot of transcriptomes from 5 day spheroid (MCS) cultures and monolayer cultures of HCT116 cells. GSEA plots are for hypoxia, glycolysis and mitosis. NES: normalized enrichment score.

(b) ER stress in HCT116 MCS. Proteins were extracted from monolayer and 5 day MCS cultures and subjected to western blotting using the indicated antibodies. Increased cleavage of caspase-4 in cells grown as spheroids shown by asterix .

(c) Glucose concentration in MCS. Different number of cells were seeded per well to generate MCS with different sizes (number of cells x 10^{-3}). Glucose was measured after 5 days. Larger MCS contain lower levels of glucose/cell. Glucose levels subsequently decreased to ~50% of these values at day 9. Shown are means $\pm$ S.D. (n = 3); t-test.

(d) Viability of MCS core areas is dependent on glucose availability. HCT116 MCS were formed in high-glucose medium, shifted to the indicated concentrations of glucose for 24 h followed by fixation and sectioning. Bar = 250 μm.

(e) Screen for compounds that induce loss of viability of HCT116 colon cancer MCS. Spheroids (5-day after formation) were treated for 6 h with 25 μM of compounds from the Chembridge DiverseSet (10,000 cpds) and viability was measured using the acid phosphatase test. Compounds inducing > 50% loss of viability were rescreened at 25, 12, 6 and 3 μM at the same conditions. This procedure finally lead to the identification of 6

compounds active at 3 μM. These 6 compounds were then tested for reduction of clonogenicity of HCT116 MCS (see Figure 1d).

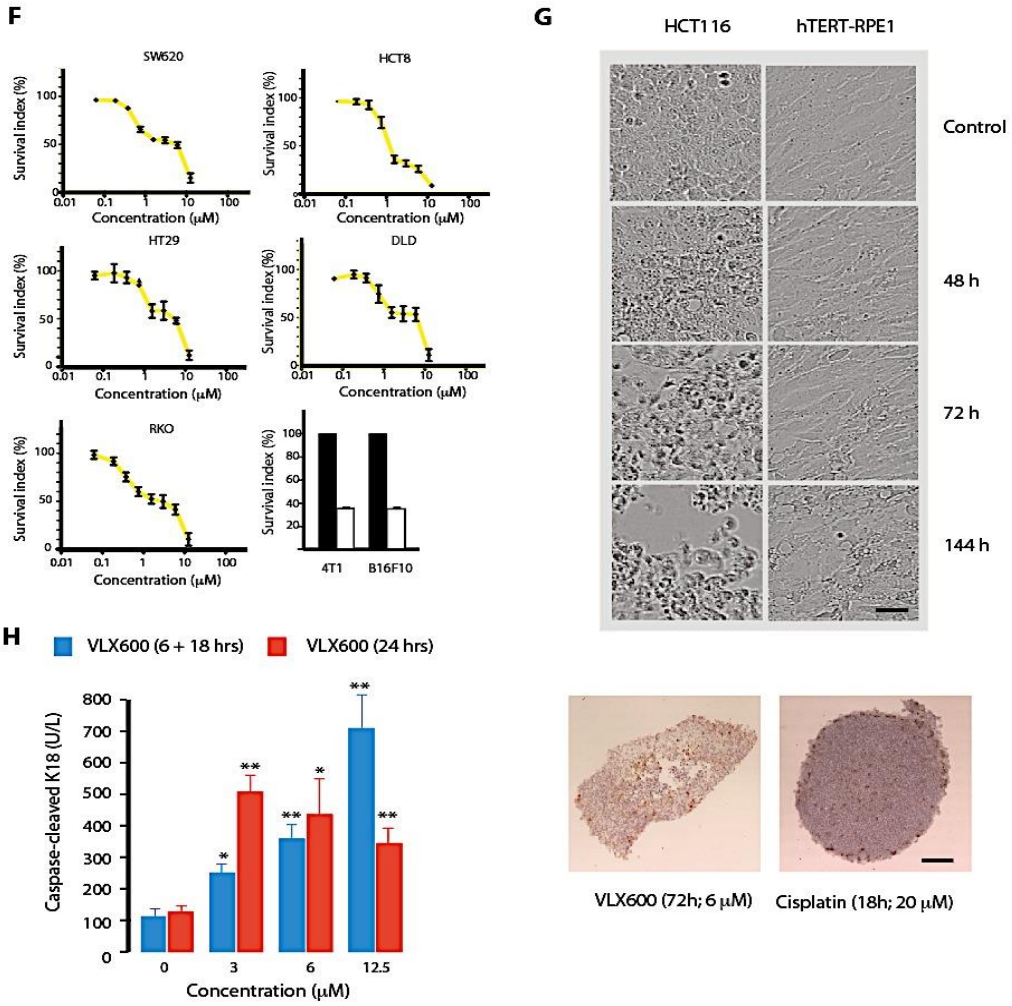

(f) Monolayer cultures of a panel of human colon cancer cell lines were incubated with the indicated concentrations of VLX600 and viability was examined after 72 hours using FMCA (Lindhagen et al., Nature Protoc. 3 (2008) 1364-9). In the lower right panel, two mouse cell lines (4T1 breast carcinoma; B16F10 melanoma) were exposed to 6 μM VLX600 and viability measured after 72 h. (n = 3; means±S.E.M)

(g) Phase contrast microscopic images of HCT116 and hTERT-RPE1 cells treated with 6 μM VLX600 for the indicated times in hours. Bar = 25 μm.

(h) (left) Induction of caspase-cleaved keratin 18 (ccK18) by VLX600. Monolayer cells were treated with the indicated concentrations of VLX600 either continuously for 24 h or for 6 h followed by medium change and incubation in drug-free medium for 18 hours. Accumulation of caspase-cleaved K18 was determined using the M30 CytoDeath® ELISA. The assay provides an integrative determination of apoptosis by analysing ccK18 in both cells and cell medium (non-ionic detergent was added to the cell medium at the end of the incubation and all content analysed). Shown are means ± S.D. (n = 3); (* $p < 0.05$, ** $p < 0.01$; t-test).

(right) Staining for active caspase-3. MCS were exposed to VLX600 (6 μM; 72 h) or cisplatin as a reference (20 μM; 18 h), sectioned and stained for active caspase-3. The diffuse staining of MCS exposed to VLX600 and the peripheral staining of MCS exposed to cisplatin. Bar = 100 μm.

I

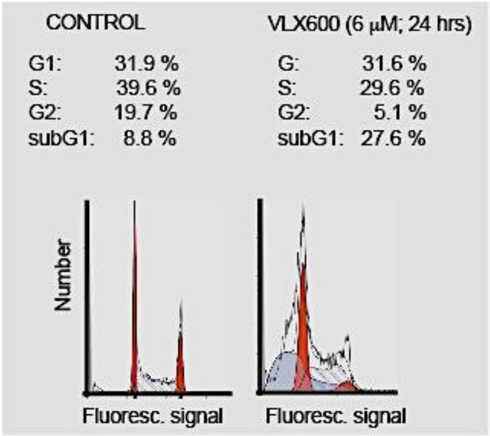

K

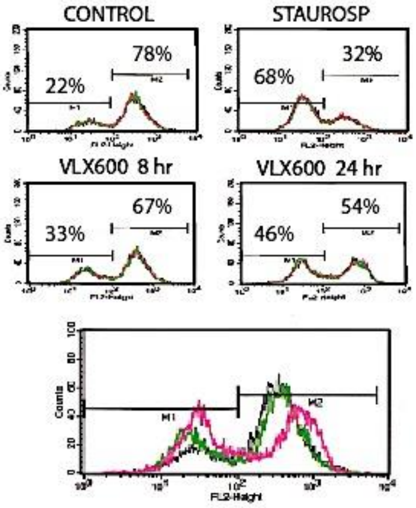

L

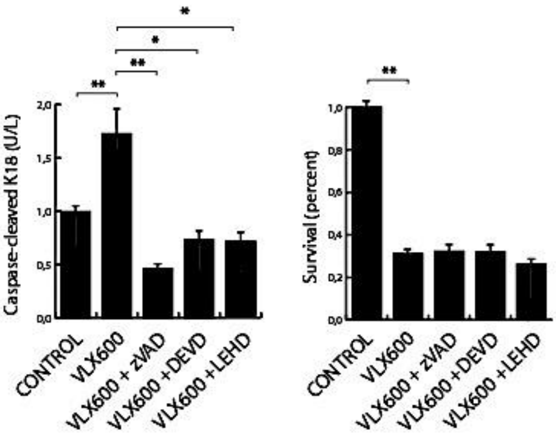

(i) Cell cycle analysis of VLX600-treated cells (6 μM, 24 hours). Cells were stained with propium iodide and analysed by flow cytometry.

(k) Analysis of mitochondrial membrane potential (Δψ) by staining with JC1 and flow cytometry. The number of cells which are weakly stained by JC1 increased from 22% in control to 46% after 24 h of treatment with VLX600). Cells were treated with 6 μM VLX600 or 1 μM staurosporine. In the lower panel the JC1 data is presented in a composite form; black = untreated; green = 8 hrs VLX600, pink = 24 hours.

(l) Inhibition of caspase-cleavage of K18, but not cell survival, by caspase inhibitors. HCT116 cells were exposed to 6 μM in the presence or absence of the indicated caspase inhibitors (10 μM). The levels of ccK18 was quantified after 24 h (left) and cell viability after 72 h (right). Statistical analysis by t-test (comparisons as indicated). The low levels of ccK18 in samples exposed to VLX600 and caspase-inhibitors is due to inhibition of spontaneous apoptosis observed in the untreated control during the incubation period. Shown are means ± S.D. (n = 3); (* $p < 0.05$, ** $p < 0.01$; t-test).

Supplementary Figure 2

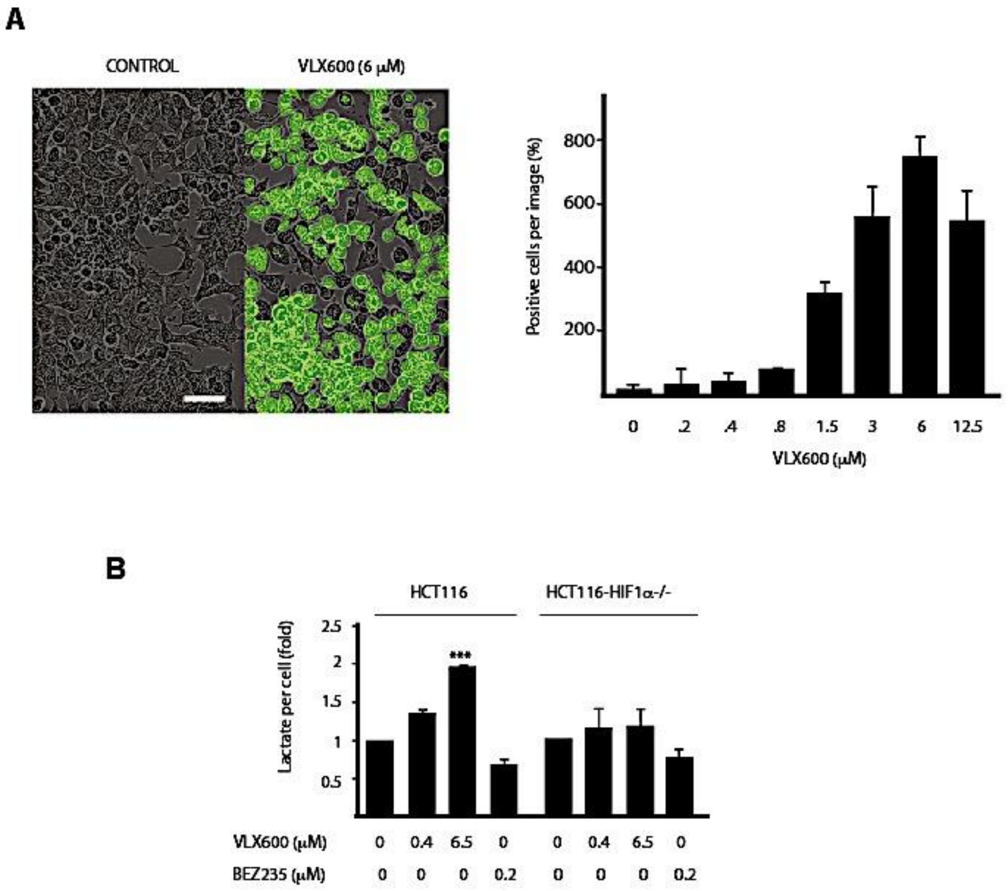

(a) Accumulation of GFP in HCT-116 HRP/EGFP cells exposed to the indicated concentrations of VLX600. This reporter cell line has been described by Ravizza *et al.* (Eur J Cancer 45 (2009) 890–8) and expresses EGFP under the control of an artificial hypoxia-responsive promoter (HRP). The number of positive cells per image is quantified to the right; Shown are means ± S.D. (n=3). Bar=100μm.

(b) Lactate levels in the medium of HCT116 or HCT116$^{HIF\text{-}1\alpha\text{-/-}}$ cells were assayed after 24 hours after treatment with VLX600 or NVP-BEZ235 at indicated concentrations. The increase of lactate between 0.4 to 6 μM is significant at $p = 0.0007$ (t-test); the small increases in HCT116$^{HIF\text{-}1\alpha\text{-/-}}$ cells were not significant. VLX600, but not the PI3K/mTOR inhibitor NVP-BEZ235, induces increased production of lactate in HCT116 cells. Shown are means ± S.D. (n = 3); (*** $p < 0.001$; t-test).

Supplementary Figure 3

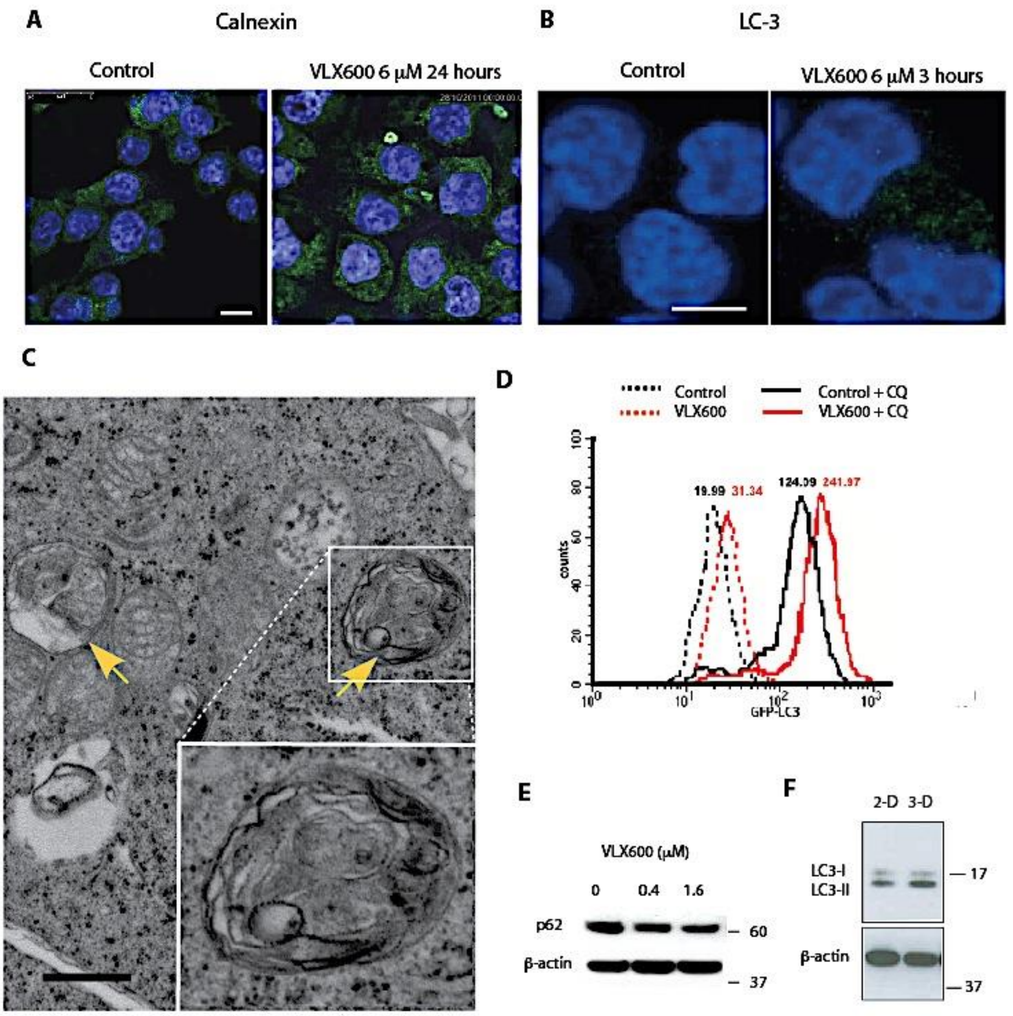

(a) HCT116 cells (VLX600-exposed and control as indicated) stained with an antibody to the ER marker calnexin. The vesicles observed in Figure 3a do not stain with the calnexin antibody. Bar = 5 μm.

(b) Confocal microscopy analysis of HCT116 cells exposed to VLX600 (6 μM, 3 h) and stained with an antibody to LC3. The increased small cytoplasmic dots are likely to be autophagosomes. Bar = 5 μm.

(c) Electron microscopy of HCT116 cells exposed to VLX600 (6 μM) for 24 h. Autophagosomes containing partially digested structures are indicated with arrows. Bar = 0.5 μm.

(d) Autophagic flux was confirmed using osteosarcoma HOS cells expressing GFP-LC3. Cells were treated with 6 μM VLX600 and 50 μM chloroquine (CQ) was added during the last 4 hours of incubation. After mild saponin extraction, autophagosomes-associated LC3 fluorescence was measured by flow cytometry (for details of the method, see Eng *et al.*, Autophagy 6 (2010) 634). The data show that VLX600 induced a significant increase in autophagic flux.

(e) Exposure to VLX600 leads to decreases in the levels of p62/SQSTM1. HCT116 cells were treated with the indicated concentrations of VLX600 for 24 hours and subjected to western blotting.

(f) Analysis of LC3 levels in HCT116 cells grown in monolayer culture or as MCS. Proteins were extracted from HCT116 cells grown in monolayer or as MCS and subjected to western blotting.

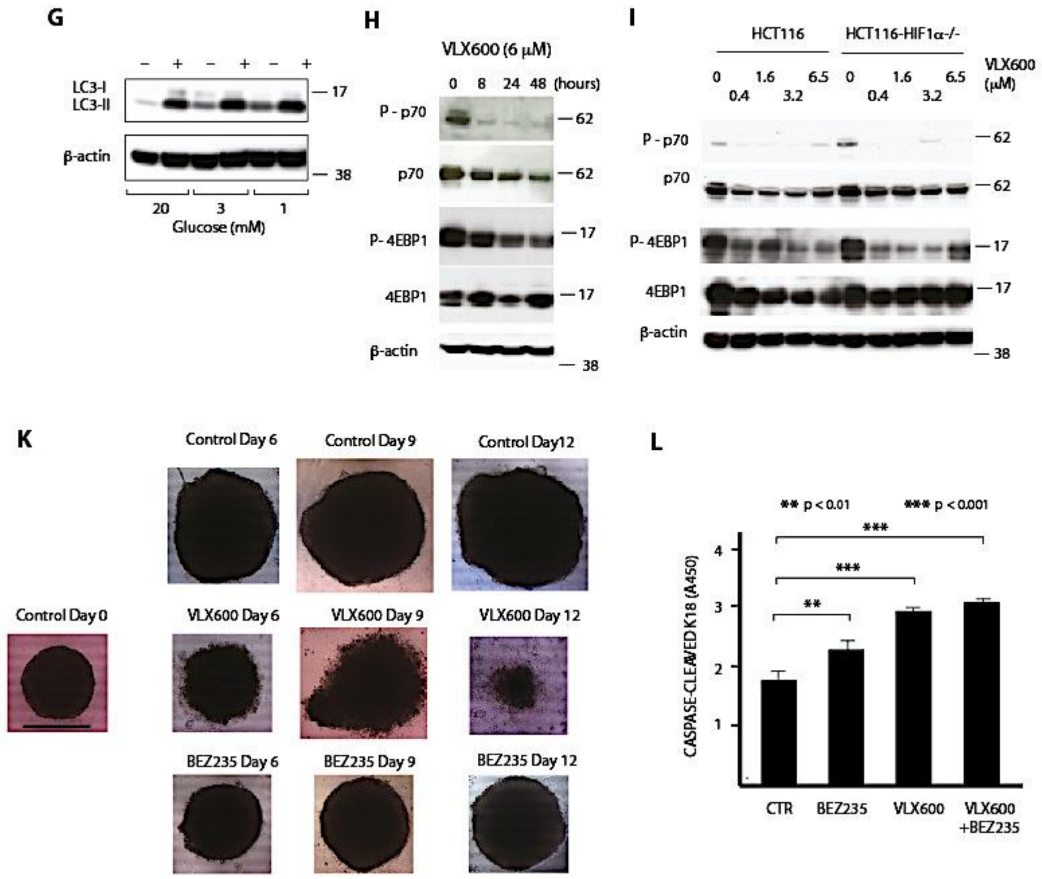

(g) Analysis of LC3 levels in HCT116 cells grown in different concentrations of glucose in monolayer culture.

(h) VLX600 decrease phosphorylation of p70 and 4EBP1. HCT116 cells were exposed to VLX600 (6 μM) for the times indicated and protein extracts were processed for western blotting.

(i) Reduction of phosphorylation of 4EBP1 and p70 by VLX600 does not require HIF1-α. HCT116^{wt} or HCT116$^{HIF\text{-}1\alpha\ -/-}$ cells were exposed to different concentrations of VLX600 for 24 hours and subjected to western blotting using the indicated antibodies.

(k) MCS were treated with the indicated compounds for 3 days, washed and incubated in drug-free medium. Photographs were taken at the times indicated. Note that VLX600-treated MCS have undistinct borders, indicative of dead cells (the collapse between day 9 and 12 is due to disintegration of dead cells). Also note that the PI3K/mTOR inhibitor NVP-BEZ235 inhibits MCS growth but does not eradicate MCS. Bar = 500 μm (in the control day 0 photograph).

(I) Apoptosis induction in HCT116 MCS by VLX600 or NVP-BEZ235. MCS were exposed for 24 h to VLX600 (6 μM) or NVP-BEZ235 (0.2 μM) and the accumulated levels of caspase-cleaved K18 were determined using M30 CytoDeath® ELISA. Shown are means ± S.D. (n = 3); statistics by t-test.

Supplementary Figure 4

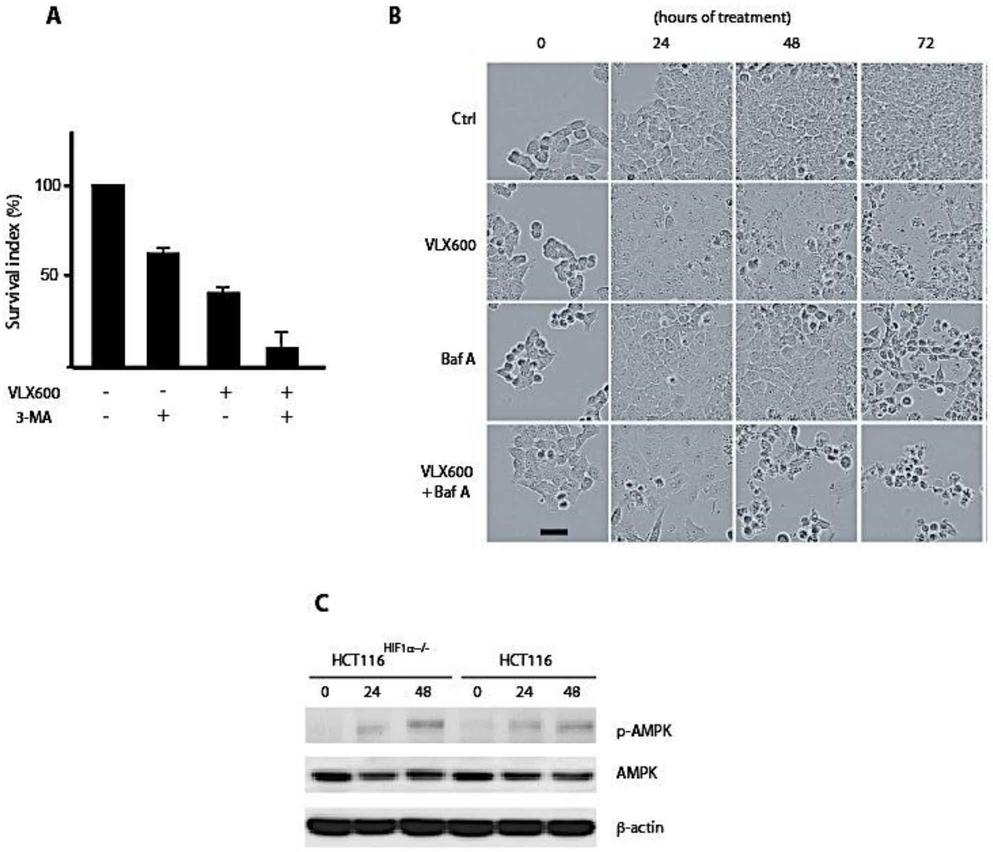

(a) HCT116 cells were treated for 48 hours with 6 μM VLX600 in the presence or absence of 3 mM 3-MA (3-methyladenine) as indicated and viability was determined by the acid phosphatase test. (n = 3, Shown are means ± S.D.).

(b) HCT116 cells were treated with 6 μM VLX600 in the presence or absence of 0.25 μM bafilomycin A as indicated and photographed under the phase contrast microscope. Bar = 100μm.

(c) HCT116 or HCT116$^{HIF-1\alpha -/-}$ cells were treated with 6 μM VLX600 for 24 or 48 hours and subjected to western blotting using antibodies to total or phosphorylated AMPK.

Supplementary Figure 5

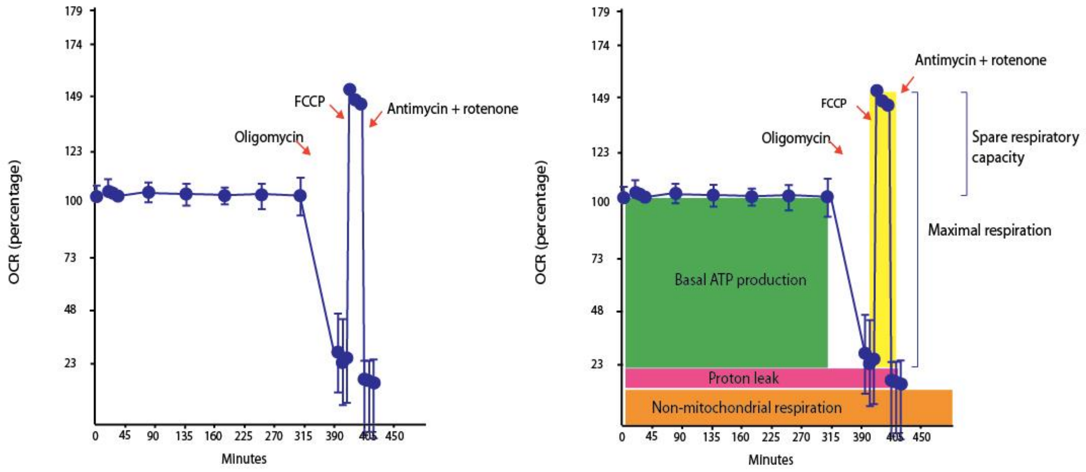

Analysis of oxygen consumption rates (OCR) in HCT116 cells using a Seahorse XF Analyzer. Oligomycin was added to the cells, followed by the uncoupler FCCP and antimycin/rotenone. Shown are means $\pm$ S.D.($n = 3$). The $\sim$ 75% decrease in oxygen consumption rates after addition of oligomycin shows that oxidative phosphorylation is responsible for the majority of oxygen consumption in this colon carcinoma cell line. A guide to the interpretation of results is provided in the right panel (showing the same data as in the left panel).

Supplementary Figure 6

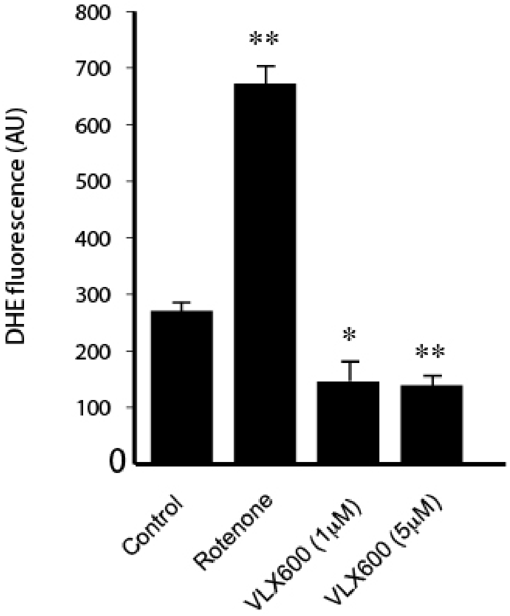

VLX600 reduces ROS levels in HCT116 cells. ROS was determined using staining with dihydroethidium after 24 h. Rotenone (10 µM), was used as a positive control. (n = 3. Shown are means ± S.D. ; * $p < 0.05$** $p < 0.01$; t-test).

Supplementary Figure 7

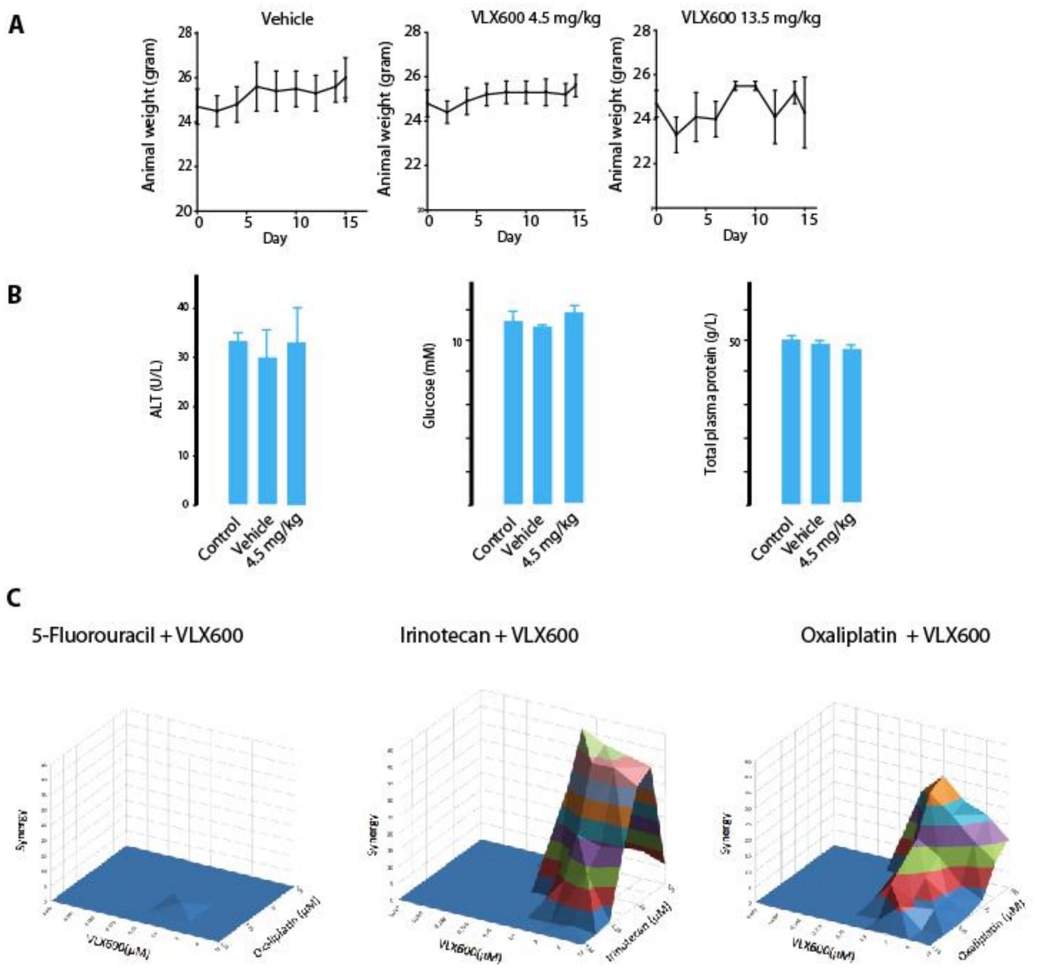

(a) Weight of animals (n = 8) treated with the indicated doses of VLX600. Means ± S.D.

(b) Plasma concentrations of ALT (alanine transaminase), glucose and total plasma protein determined at the time of sacrifice of animals. Means ± S.D.(n = 8).

(c) Effect of combination therapy. VLX600 was used to treat HCT116 cells in combination with 5-fluorouracil, irinotecan or oxaliplatin for 72. Viability was tested using FMCA. In these graphical illustrations (using MacSynergy™ II (Pritchard, Aseltine and Shipman, Univ of Michigan)), additive effects are shown as a flat surface, synergies as "mountains" and antagonistic effects as "valleys".

Supplementary Figure 8

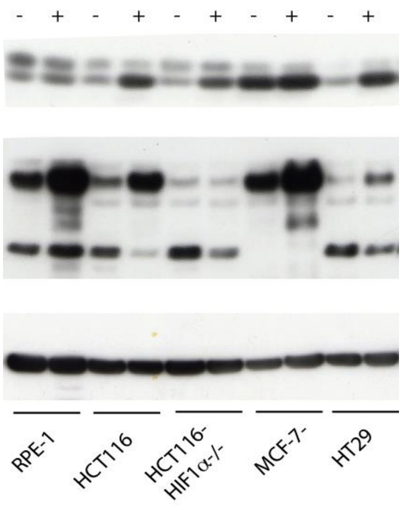

LC-3

BNIP3

β-actin

Uncropped images of Fig 4a. MCF-7 is a breast cancer cell line and these slots were removed in Fig. 4a.

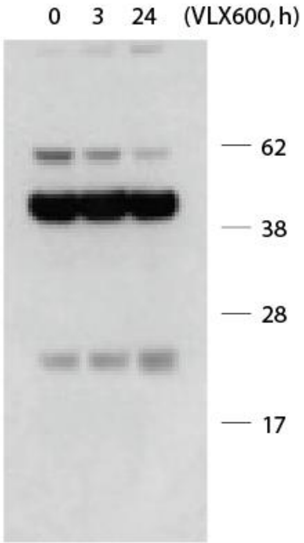

COX-1

β-actin

Cross-reacting material

Immunoblot probed both for COX-1 and β-actin (Fig. 5e).

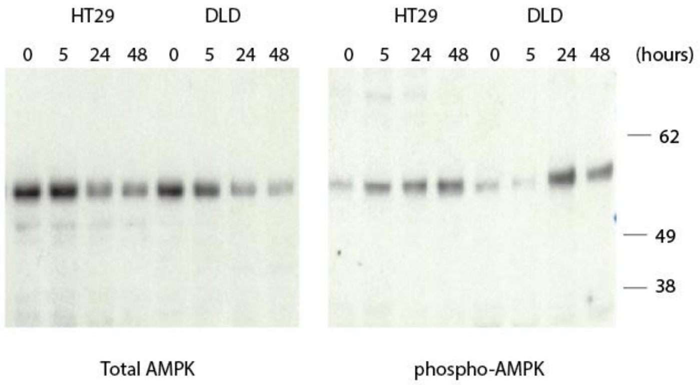

Key experiment showing induction of AMPK phosphorylation in colon cancer cells after exposure to 6 μM VLX600.
All slots are shown in Fig. 4d.

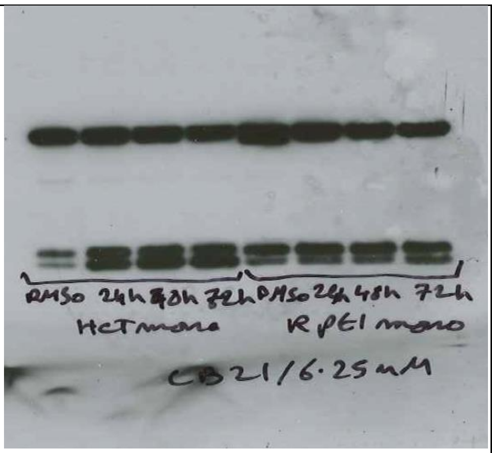	Immunoblot probed both for LC3 (lower bands) an β-actin (upper band). The left four slots are shown in Fig. 3D. VLX600 (=CB21) induces LC3-II in HCT116 cells grown as monolayer culture (left 4 slots) but not in untransformed RPE1 cells (right 4 slots).	
